# Synthesis, antioxidant and antimicrobial activities, molecular docking study of new pyrimidine derivatives

**DOI:** 10.1038/s41598-026-45654-3

**Published:** 2026-04-13

**Authors:** Hemat S. Khalaf, Ahmed A. El-Rashedy, Amina A. Abd El-Gwaad, Ahmed A. Fayed

**Affiliations:** 1 Photochemistry Department, National Research Centre, Chemical Industries Research Institute, 33 El Buhouth Street, P.O. Box 12622, Cairo, Egypt; 2 Natural and Microbial Products Department National Research Centre, Pharmaceutical and Drug Industries Research Institute, 33 El Buhouth Street, P.O. Box 12622, Cairo, Egypt; 3 Department Organic and Medicinal Chemistry, Faculty of Pharmacy, University of Sadat City, Cairo, 32897 Menoufia Egypt; 4 Therapeutic Chemistry Department, National Research Centre, Dokki, P.O. Box 12622, Cairo, Egypt

**Keywords:** Pyrimidine, Thiazolopyrimidine, Triazolopyrimidine, Chalcone, Oxirane pharmacological, Docking, Antioxidants, Antimicrobials, Biochemistry, Biotechnology, Chemistry, Computational biology and bioinformatics, Drug discovery, Microbiology

## Abstract

Organic compounds, especially heterocyclic compounds, are known for their significant bioactivity, in this work, novel pyrimidine derivatives **3–11** were synthesized from the reaction of chalcon compound **1** with hydrogen peroxide to give oxiran compound **2** which reacted with thiourea producing the precursor key compound **3**. Elemental analysis and several spectroscopic techniques were used to determine the molecular structures of the novel derivatives. All the new analogs were screened against four human pathogenic microbial strains: Escherichia coli as Gram-negative bacteria, Bacillus subtilis as Gram-positive bacteria and the yeast Candida albicans and Aspergillus niger using agar diffusion method, suggesting broad-spectrum potential. whereas compounds **5, 9a** and **11** exhibited high inhibitory action with an inhibition zone of 17–20 mm compared to standard drugs, Streptomycin and Cycloheximide. Antioxidant profile employing DPPH radical scavenging studies further proved their capacity to lower oxidative stress. Whereas compounds **5, 9a** and **11** showed significant DPPH activity and antioxidant activities. Additionally, at doses of 2.0, 1.0, and 0.5 mg/ml, compounds **3, 4, 6a, 8** and **9b** showed moderate antioxidant activity. Conversely, compounds **6b, 6c, 7** and **10** showed very little DPPH activity. Molecular docking tests revealed strong binding affinities with significant microbial enzymes, which corroborated these conclusions and were in line with their observed inhibitory effects. Complementary and drug-like in silico ADMET testing confirmed that Lipinski’s recommendations were followed, showing that oral delivery was suitable. Some of the produced compounds showed good pharmacological activity when compared to reference controls. A comprehensive synthesis, spectroscopic analyses, and pharmacological activity were all reported.

**Supplementary Information:**

The online version contains supplementary material available at 10.1038/s41598-026-45654-3.

## Introduction

Modern medicine continues to face a significant challenge from the ongoing evolution of antibiotic-resistant bacterial species^1^. Compromising the effectiveness of numerous broad-spectrum and first-line medicines. The rising rates of resistance displayed by Staphylococcus aureus and E. coli, two major sources of severe infections worldwide, are especially concerning^2^. 

In the past two decades, invasive fungal infections have also increased, especially in immunosuppressed individuals, becoming a leading cause of illness and death. Recently, medicinal chemists have focused on developing drugs with improved efficacy, longer action, and reduced toxicity^3^. Pyrimidine derivatives are widely recognized in medicinal chemistry for their therapeutic uses. Pyrimidine compounds are vital building blocks for drugs that produce antiviral^4^, antioxidant^5^, anticancer^6,7^, They also display a broad spectrum of biological actions, including antineoplastic^8^, antihistaminic^9^, analgesic, anti-inflammatory, neuroprotectants, and anti-neuroinflammatory agents^10–12^. According to reports, condensed pyrimidine derivatives have antihypertensive^13^, and central nervous system effects^14^. Also, pyrimidine have previously reported as dual EGFR/VGFR2 inhibitors^15,16^, antihypertensive^17,18^, antitubercular^19^, antibacterial^20,21^, antimalarial^22^, antibiofilm^23^, analgesic^24^, and anti-HIV^25^. Additionally, pyrimidine rings are present in a variety of natural substances, including vitamin B1 (thiamine), as well as a number of synthetic compounds, including vernal and barbituric acid^26^. Molecular docking is widely used to predict how small pharmaceuticals will interact with their protein targets, providing crucial details on the compounds’ activity and affinity. The rational development of pharmaceuticals depends on this process. Due to the biological and pharmacological importance of docking studies, a great deal of effort has been made to improve algorithms for accurate docking predictions^27^. Docking is essential for assessing how synthetic compounds interact with protein receptors because it offers vital information about the medicines’ binding processes and potential biological properties^28,29^. In this regard, the present work is concerned with designing and synthesizing various novel poly heterocyclic fused ring systems including thiopyrimidine in view of these observations and we have evaluated their antioxidants and antimicrobials activities in comparison to reference drugs, aiming to identify candidates with promising dual-function bioactivity.

## Results and discussion

### Chemistry

**Scheme 1:**
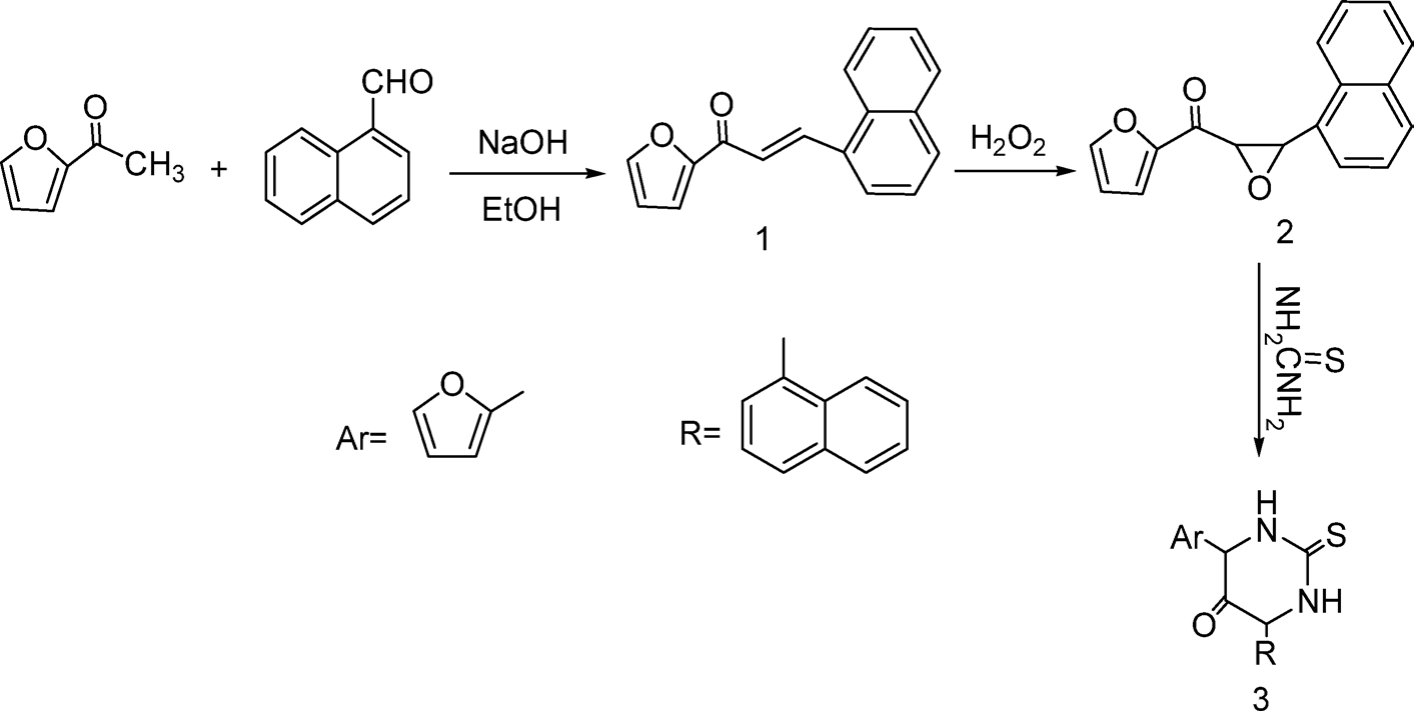
Synthetic route for compounds **1–3**.

Chalcone **1** was produced by reacting 2-acetylfuran with naphthalene-2-aldehyde, in an alkaline medium. Reaction of chalcone **1** with hydrogen peroxide afforded oxiran derivatives **2,** where 1666 cm^−1^(C=O) of chalcone shifted to 1672 cm^−1^ in IR spectrum, the appearance of this peaks 4.13–4.16 (d, J = 15 Hz, 1H, epoxy-H beta to C=O), 4.41–4.44 (d, J = 15 Hz, 1H, epoxy-H alpha to C=O) in ^1^H NMR spectrum (CDCl3, δ ppm), Compound **2** reacted with thiourea in ethanolic potassium hydroxide to give pyrimidine derivatives **3** as starting material (Scheme 1).

**Fig. 1:**
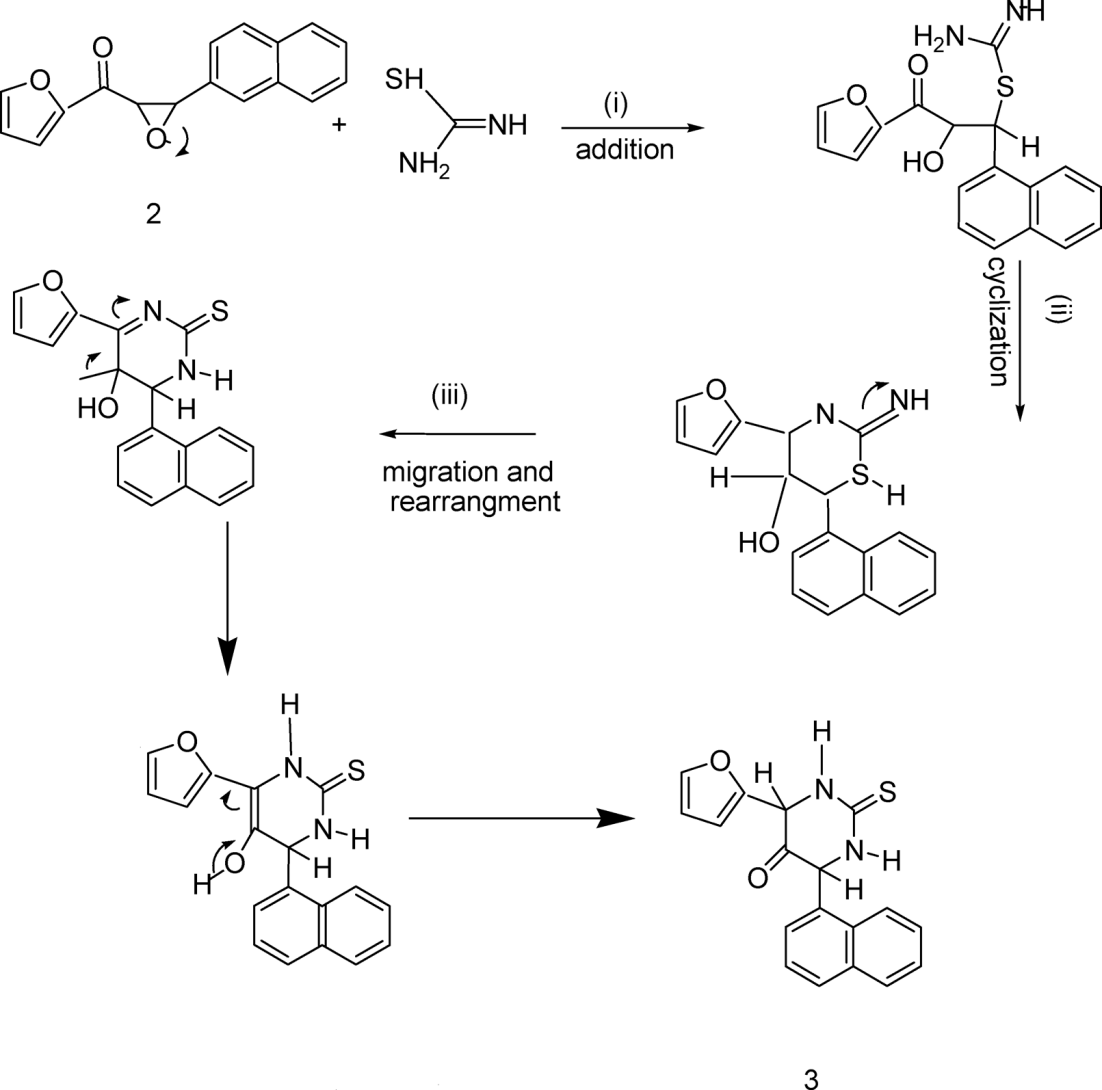
Mechanism to synthesis of compound 3.

IR spectrum of compound **3** showed signals for (NH) and (C=S), and Fig. 1 illustrates mechanism to form compound **3**.

**Scheme 2:**
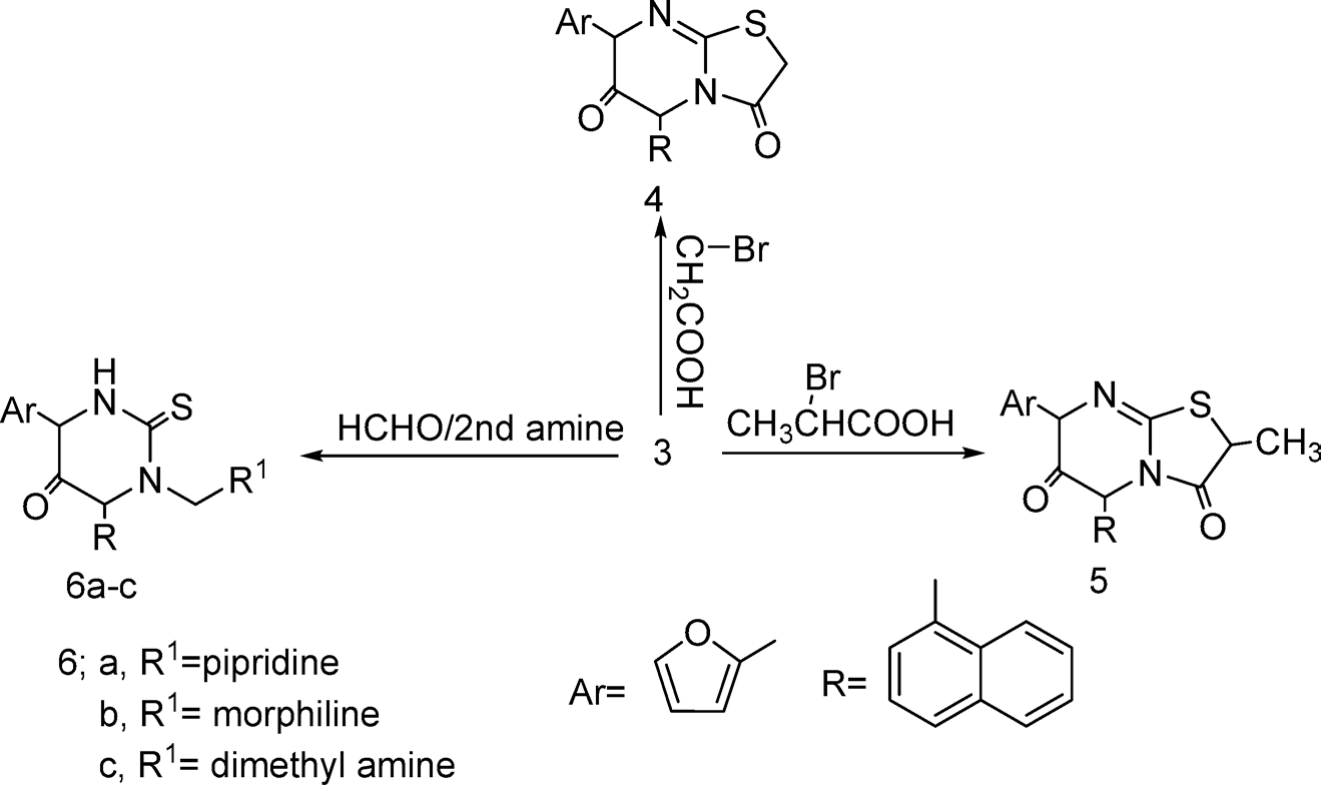
Synthetic route for compounds **4-6a-c.**

**Fig. 2:**
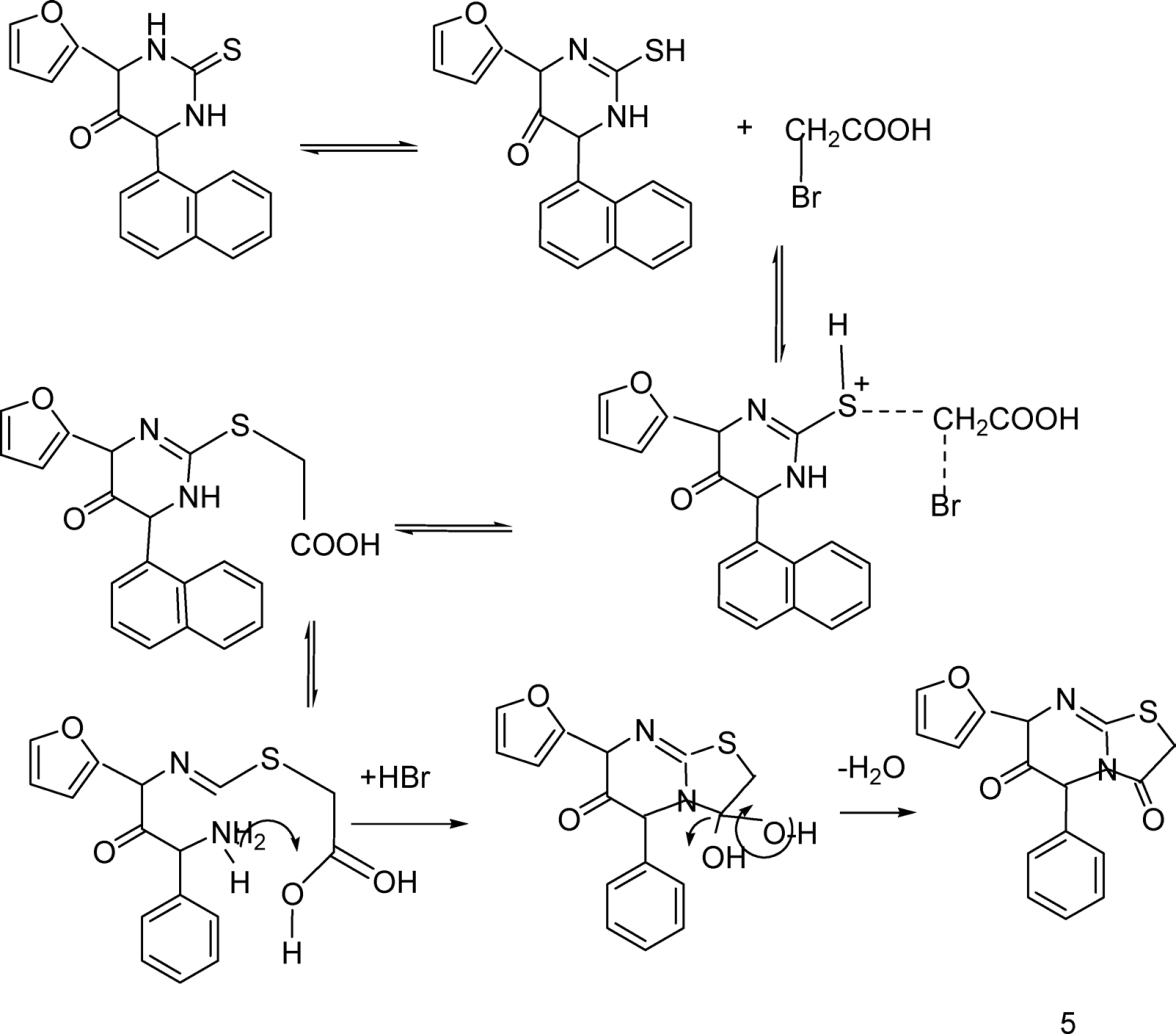
Mechanism to synthesis of compounds **4** and **5**.

Reaction of pyrimidine derivatives **3** with bromoacetic acid and/or 2-bromopropionic acid respectively, afforded thiazolopyrimidine derivatives **4** and **5** respectively. IR spectrum showed disappearance of signals of (NH) and appearance tow signals for (C=O), Also, ^1^H NMR spectrum shows signal of methyl group. Also, pyrimidine derivatives **3** reacted with formaldehyde and secondary amine namely, piperidine, morpholine and/or dimethyl amine respectively, to afford Mannich bases **6a-c** respectively, ^1^H NMR spectrum shows signal of methylen group (N-CH_2_-N). (Scheme 2). Over the years there has been controversy about the mechanism of the Mannich reaction, especially whether the aldehyde is first attached by active hydrogen compound or by the amine. It is now generally agreed that the latter path way is the correct one (Fig. 2).

**Fig. 3:**
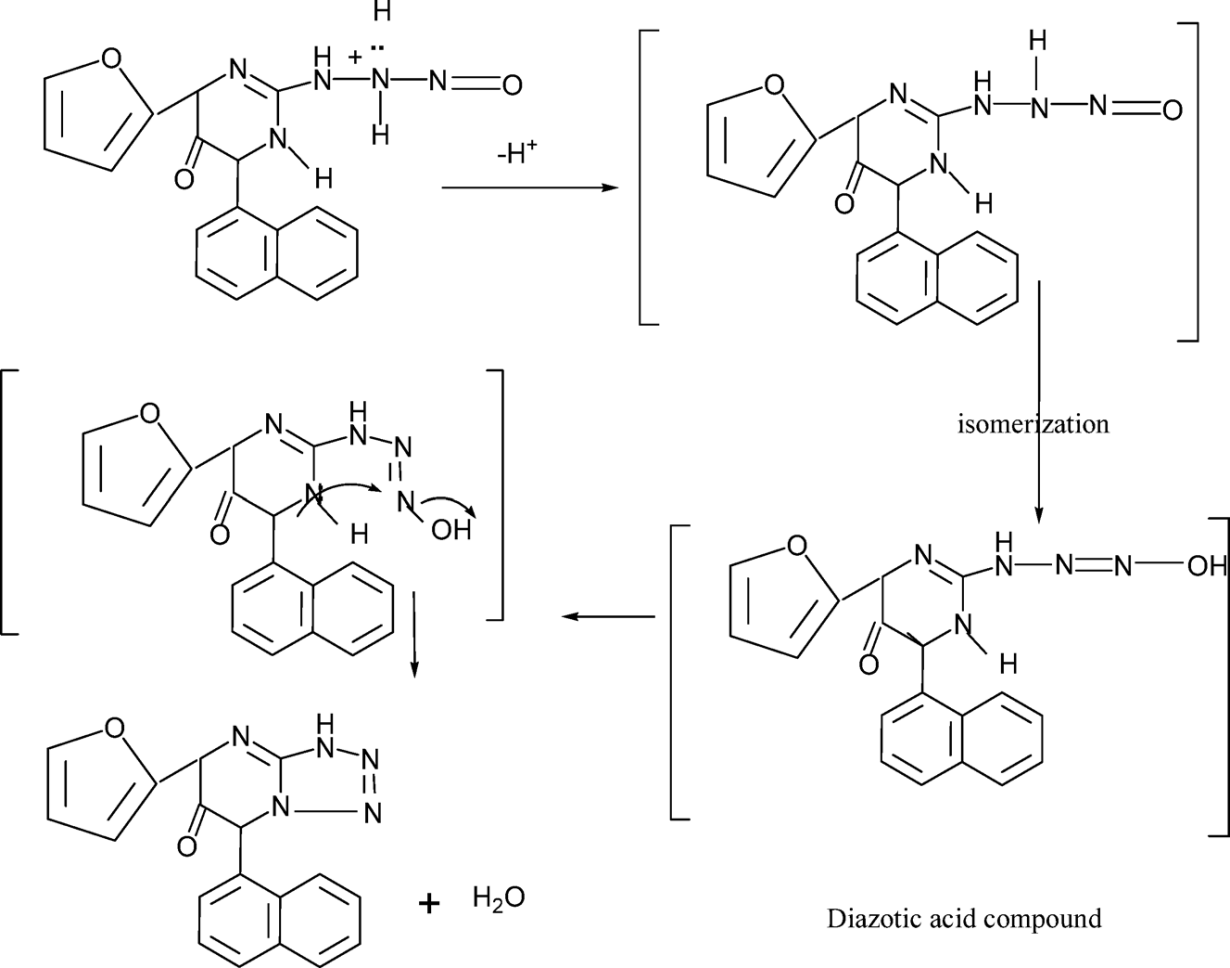
Mechanism to synthesis of compound **8**.

**Fig. 4:**
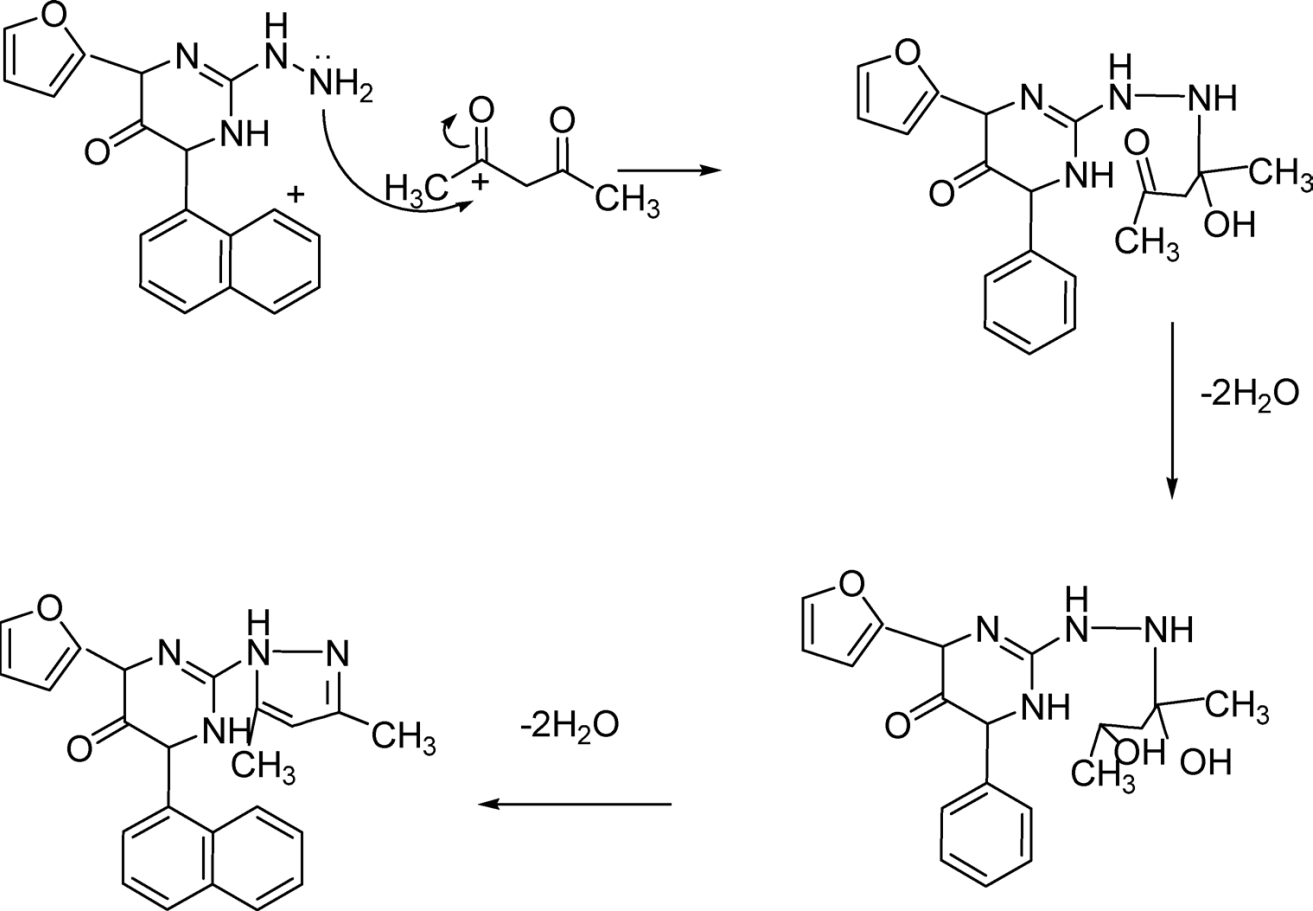
Mechanism to synthesis of compound **10**.

**Scheme 3:**
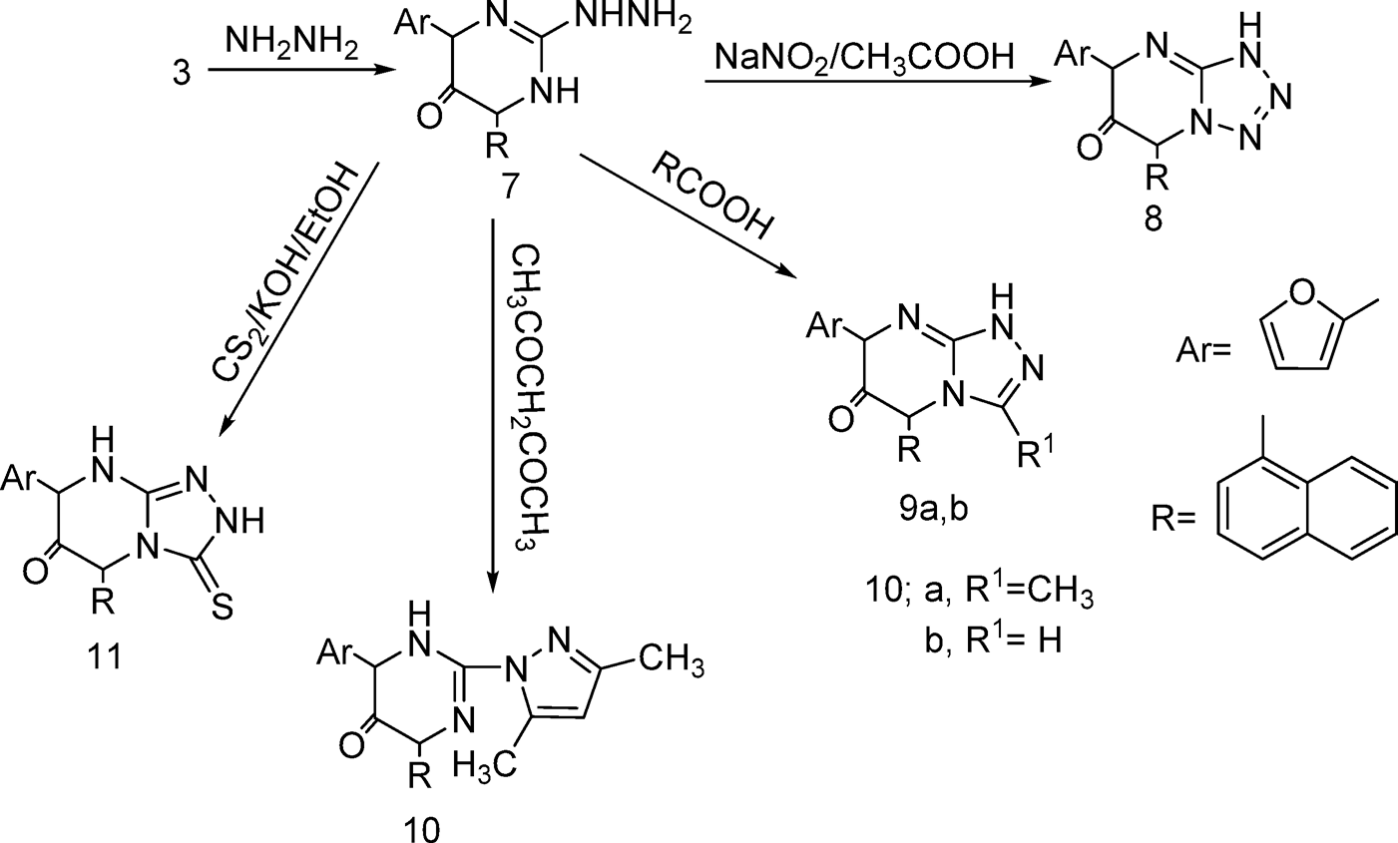
Synthetic route for compounds **7–11**.

Treatment of compound **3** with hydrazine hydrate afforded the corresponding hydrazinopyrimidine derivatives **7**. Diazotization of compound **7** in presence of glacial acetic and sodium nitrite afforded tetrazolopyrimidine derivatives **8**. N-nitrosoamine formed in the reaction of the primary amine compound **7** with nitrous acid is unstable and isomerizes by a process analogous to interconversion of a ketone to an enol. The prouduct is called a diazotic acid compound R–N=N–OH which cyclized led to the formation of tetrazolo compound **8 **(Fig. 3). Also, compound **7** reacted with acetic acid and /or formic acid, respectively to afford triazolopyrimidine derivatives **9a, b** respectively. Further condensation of compound **7** with acetyl acetone in absolute ethanol gave diazolopyrimidine derivatives **10 **(Fig. 4). Finally, compound 7 reacted with carbon disulphide in ethanolic potassium hydroxide solution to afford triazolopyrimidine derivatives **11** (Scheme 3).

### Biological activities

Two *Biological* activities were examined: antioxidants and antimicrobials. Compounds 3–11 were examined for their antioxidants and antimicrobials properties. Depending on their structure and function groups, these chemicals have varying actions.

#### Antioxidant activities

The antioxidant capacity of the synthesized compounds was assessed using a variety of tests, such as DPPH, and compared to a positive control of butylated hydroxytoluene (BHT) at a concentration of 50 µg/ml. At concentrations of 2.0, 1.0, and 0.5 mg/ml, compounds (5, 9a and 11) showed significant DPPH activity and antioxidant activities ranging from (31.40 ± 0.11, 32.30 ± 0.13 and 34.80 ± 0.12) to (17.32 ± 0.11, 15.15 ± 0.11 and 18.28 ± 0.13). Additionally, at doses of 2.0, 1.0, and 0.5 mg/ml, compounds (3, 4, 6a, 8 and 9b) showed moderate antioxidant activity ranging from 28.88 ± 0.11, 25.80 ± 0.12, 20.10 ± 0.11, 24.80 ± 0.12 and 23.80 ± 0.11) to (13.55 ± 0.13, 11.50 ± 0.12, 8.50 ± 0.12, 14.22 ± 0.12 and 12.50 ± 0.12). Conversely, compounds (6b, 6c, 7 and 10) showed very little DPPH activity, with DPPH values of (18.55 ± 0.11, 16.80 ± .0.13, 18.50 ± 0.12 and 19.80 ± 0.11) (7.40 ± .0.11, 5.18 ± 0.0.13, 8.30 ± 0.13 and 10.50 ± 0.11) at 2.0 and 1.0 mg/ml, respectively. (Table 1). These substances exhibit redox characteristics that contribute to their antioxidant qualities by allowing them to act as reducers, suppliers of hydrogen atoms, and scavengers of free radicals. These compounds exhibit antioxidant capabilities either by electron contribution or by partnering the DPPH radical with a hydrogen atom. These results are consistent with the work of Khalaf et al., who similarly used DPPH scavenging experiments to assess the antioxidant capacity of synthesized compounds. Furthermore, Khalaf et al.^28^ emphasized that the substituents on the phenyl ring affect the antioxidant efficacy of such compounds, with a focus on the significance of hydroxyl groups in improving antioxidant performance.

**Table 1:** Antioxidant activity of synthesized compounds 3–11.

Compounds NO.	Antioxidant Activity DPPH%		
	2.0 mg/ml	1.0 mg/ml	0.5 mg/ml
Control (BHT)	65.34 ± 0.13	34.27 ± 0.15	22.52 ± 0.12
3	28.88 ± 0.11	13.55 ± 0.13	0.0
4	25.80 ± 0.12	11.50 ± 0.12	0.0
5	31.40 ± 0.11	17.32 ± 0.11	0.0
6a	20.10 ± 0.11	8.50 ± 0.12	0.0
6b	18.55 ± 0.11	7.40 ± 0.11	0.0
6c	16.80 ± .0.13	5.18 ± .0.13	0.0
7	18.50 ± 0.12	8.30 ± 0.13	0.0
8	24.80 ± 0.12	14.22 ± 0.12	0.0
9a	32.30 ± 0.13	15.15 ± 0.11	0.0
9b	23.80 ± 0.11	12.50 ± 0.12	0.0
10	19.80 ± 0.11	10.50 ± 0.11	0.0
11	34.80 ± 0.12	18.28 ± 0.13	0.0

*The experiment was carried out in triplicate. Values are given as mean ± standard error.

*Butylated hydroxytoluene (BHT).

#### The IC_50_ value of DPPH radical scavenging activity

The concentration of the sample needed to inhibit 50% of radicals was determined using the IC_50_ value. Compound **11** had the highest antioxidant activity (33.11 ppm), followed by **9a** (31.12 ppm) and **5** (28.11 ppm), according to the reported IC50 value (Table 2). Interestingly, control (45.13 ppm) has a higher IC_50_ value than 11.

**Table 2:** IC_50_ value of DPPH radical scavenging activity of compounds 5, 9a and 11.

Compounds NO.	IC_50_ (ppm)
5	28.11
9a	31.12
11	33.11
Control	45.13

#### Antimicrobial activity

Three samples’ antibacterial properties were assessed in vitro against *Escherichia coli* (*E. coli*) and *Bacillus subtilis (B. subtilis)*. The standard antimicrobial drug was streptomycin (10 µg). Most of the compounds had respectable antibacterial activity against the majority of the pathogens tested, according to the results in Table 3. Compounds 7 and 8 showed week inhibitory action for the gram-positive bacteria *B. subtilis*, with inhibition zones (IZ) of 6–9 mm when compared to the used standards. Compounds 3, 4, 6a, 6b, 6c, 9b and 10 showed moderate inhibitory activity with inhibition zones ranging from 12 to 15 mm in comparison to the standards used, as indicated in Table 3, whereas compound 5, 9a and 11 exhibited high inhibitory action with an inhibition zone of 18–22 mm. Compounds 7 and 8 showed week inhibitory action for the gram-negative bacteria *E. coli*, with inhibition zones (IZ) of 7–9 mm when compared to the used standards. Compounds 3, 4, 6a, 6b, 6c, 9b and 10 showed moderate inhibitory activity with inhibition zones ranging from 12–16 mm in comparison to the standards used, as indicated in Table 3, whereas compound 5, 9a and 11 exhibited high inhibitory action with an inhibition zone of 18–20 mm.

**Table 3:** The antimicrobial activity of the tested compounds 3–11.

Compounds NO.	Gram-positive bacteria	Gram-negative bacteria	Fungi	
	*B. subtilis*	*E. coli*	*A. niger*	*C. albicans*
3	15	16	15	14
4	14	12	14	13
5	19	20	18	19
6a	12	13	12	14
6b	13	13	14	13
6c	12	13	12	14
7	9	9	8	10
8	6	7	9	6
9a	18	18	17	18
9b	14	14	13	15
10	14	13	15	14
11	22	20	20	20
Streptomycin^a^	22	20	22	22
Cycloheximide^b^	20	20	22	20

^a^Streptomycin (10 µg) and ^b^Cycloheximide (50 µg).

The majority of the investigated drugs showed compounds 7 and 8 showed week inhibitory action for *A. niger*, 8–9 mm when compared to the used standards. Compounds 3, 4, 6a, 6b, 6c, 9b and 10 showed moderate inhibitory activity with inhibition zones ranging from 12 to 15 mm in comparison to the standards used, as indicated in Table 3, whereas compound 5, 9a and 11 exhibited high inhibitory action with an inhibition zone of 17–20 mm. Also, compounds 7 and 8 showed week inhibitory action for *C. albicans*, with inhibition zones (IZ) of 6–10 mm when compared to the used standards. Compounds 3, 4, 6a, 6b, 6c, 9b and 10 showed moderate inhibitory activity with inhibition zones ranging from 13 to 15 mm in comparison to the standards used, as indicated in Table 3, whereas compound 5, 9a and 11 exhibited high inhibitory action with an inhibition zone of 18–20 mm.

### Molecular dynamic and system stability

To transcend the static snapshot provided by molecular docking and evaluate the stability and dynamic behavior of the synthesized compound **11** within the DNA gyrase binding site, a comprehensive 80 ns molecular dynamics (MD) simulation was performed^30,31^. MD simulations are critical for assessing the temporal stability of ligand-receptor complexes, probing conformational flexibility, and validating docking poses under near-physiological conditions. The stability of the simulated system was rigorously evaluated using four key metrics: Root-Mean-Square Deviation (RMSD), Root-Mean-Square Fluctuation (RMSF), Radius of Gyration (Rg), and Solvent Accessible Surface Area (SASA).

**Fig. 5:**
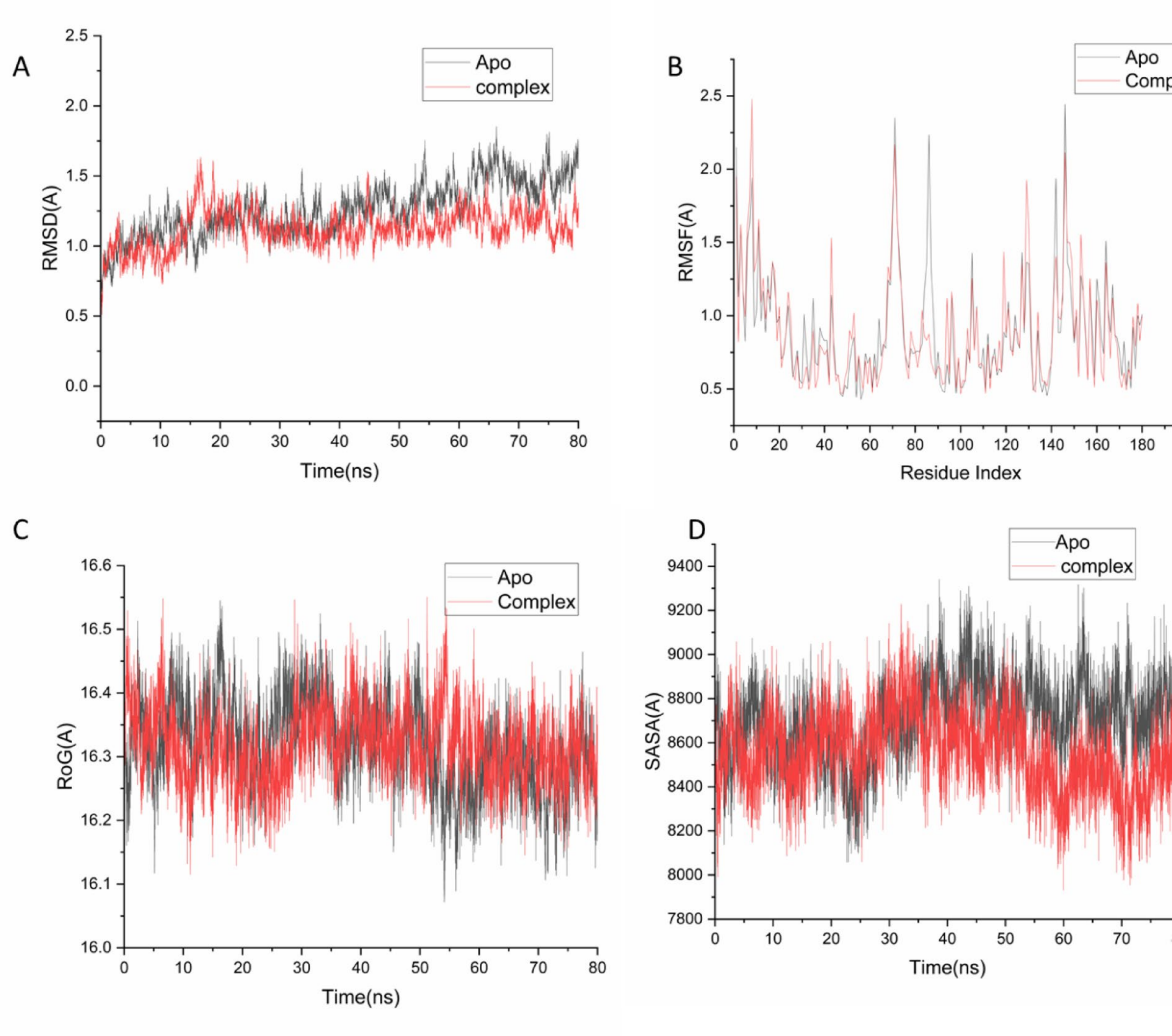
(**A**) RMSD of Cα atoms of the protein backbone atoms. (**B**) RMSF of each residue of the protein backbone Cα atoms of protein residues (**C**) ROG of Cα atoms of protein residues; (**D**) solvent accessible surface area (SASA) of the C α of the backbone atoms relative (black) to the starting minimized over 80 ns for the ATP binding site of DNA gyrase receptor with compound 11 (red).

The **RMSD** of the protein’s Cα atoms (Fig. 5A) measures the overall structural drift from the initial coordinates. The complex of DNA gyrase with compound **11** exhibited a lower average RMSD (1.12 ± 0.13 Å) compared to the unbound, Apo protein (1.26 ± 0.19 Å). This significant reduction in backbone deviation indicates that the binding of compound **11** imposes a stabilizing effect on the protein structure, constraining its global motion. A stable, low-RMSD trajectory is a hallmark of a well-bound ligand that induces minimal structural strain, a characteristic consistently observed for potent inhibitors that form stable complexes with their targets.

**Residue-specific flexibility** was analyzed via **Root-Mean-Square Fluctuation (RMSF)**^32^ (Fig. 5B). The protein in complex with compound **11** showed a lower average RMSF (0.85 ± 0.35 Å) than the Apo form (0.90 ± 0.37 Å), particularly in the binding site region. The suppression of fluctuations in key binding residues (e.g.,

Thr 128, Thr 133, Val 135) upon ligand binding suggests that compound **11** effectively occupies and engages the active site, reducing its inherent flexibility. This “locking” of the binding pocket residues is a critical feature for effective inhibition, as it prevents the conformational rearrangements necessary for the enzyme’s catalytic function^33^.

The **Radius of Gyration (Rg)**, which measures the overall compactness of the protein, further confirmed the stabilizing role of compound **11**^34,35^ (Fig. 5C). The ligand-bound complex maintained a lower and more stable average Rg (16.00 ± 0.05 Å) than the Apo protein (16.32 ± 0.06 Å). This decrease in Rg signifies a more compact and tightly folded protein structure upon inhibitor binding. Reduced compactness often correlates with enhanced structural integrity and is a strong indicator of a stable protein–ligand complex with optimal surface complementarity^36^.

Finally, the **Solvent Accessible Surface Area (SASA)** analysis^37^ (Fig. 5D) revealed that the complex had a lower average SASA (8551.7 Å^2^) compared to the Apo system (8705.43 Å^2^). A reduction in SASA, particularly for a protein–ligand complex, indicates successful burial of hydrophobic surfaces and improved packing at the binding interface. This effective shielding from solvent is energetically favorable and is a key driver of binding affinity, as it maximizes van der Waals contacts and minimizes unfavorable polar solvation.

**In summary**, the convergence of all four-stability metrics lower RMSD, suppressed RMSF at the binding site, reduced Rg, and decreased SASA—paints a coherent picture of a highly stable and well-integrated complex. Compound **11** does not merely bind but actively stabilizes the DNA gyrase ATP-binding site into a more rigid and compact conformation. This induced stability is a strong computational predictor of sustained target engagement and is in excellent agreement with the compound’s superior experimental antimicrobial profile, providing a dynamic rationale for its potency.

### Binding interaction mechanism based on binding free energy calculation

The molecular mechanics energy technique (MM/GBSA) is a prevalent method for determining the free binding energies of small molecules to biological macromolecules, using generalized Born and surface area continuum solvation, and it may provide enhanced dependability compared to docking scores^38^. The MM-GBSA tool in AMBER18 was employed to compute the binding free energies by extracting snapshots from the system trajectories. Table 4 illustrates that all reported computed energy components, with the exception of ΔG solv, had significantly negative values, signifying advantageous interactions.

**Table 4:** Shows the calculated energy binding for the synthesized compound 11 against the catalytic binding site of DNA gyrase receptor.

Energy components (kcal/mol)					
Complex	ΔE_vdW_	ΔE_elec_	ΔG_gas_	ΔG_solv_	ΔG_bind_
Compoundxx	− 46.96 ± 0.20	− 7.95 ± 0.29	− 54.92 ± 0.32	22.06 ± 0.23	− 32.85 ± 0.26

∆EvdW = van der Waals energy; ∆Eele = electrostatic energy; ∆Gsolv = solvation free energy; ∆Gbind = calculated total binding free energy.

The MM/GBSA binding free energy calculations performed with AMBER18 provide a rigorous post-docking assessment of the interaction between the synthesized Compound 11 and the DNA gyrase catalytic site, offering enhanced reliability over simple docking scores. As detailed in Table 4, the analysis reveals a highly favorable binding profile. The total binding free energy (ΔGbind = − 32.85 ± 0.26 kcal/mol) is strongly negative, driven by exceptionally favorable gas-phase interactions, which are summarized by a large negative ΔGgas value (− 54.92 ± 0.32 kcal/mol). This gas-phase stability originates from dominant van der Waals forces (ΔEvdW = − 46.96 ± 0.20 kcal/mol) and a supplementary electrostatic contribution (ΔEelec = − 7.95 ± 0.29 kcal/mol), indicating excellent shape complementarity and specific polar contacts within the binding pocket. The primary opposing force is the substantial desolvation penalty (ΔGsolv = 22.06 ± 0.23 kcal/mol), a common energetic cost associated with stripping water molecules from the ligand and receptor interface upon complex formation. Despite this solvation penalty, the magnitude of the favorable intermolecular interactions overwhelmingly compensates, resulting in a net binding affinity that strongly suggests Compound 11 is a promising and potent inhibitor of DNA gyrase.

### Identification of the critical residues responsible for ligands binding

To further investigate the key residues responsible for the inhibition of human DNA gyrase by compound 11, a per-residue decomposition of the total binding energy was performed. This analysis identifies the individual contribution of specific amino acids within the active site.

**Fig. 6:**
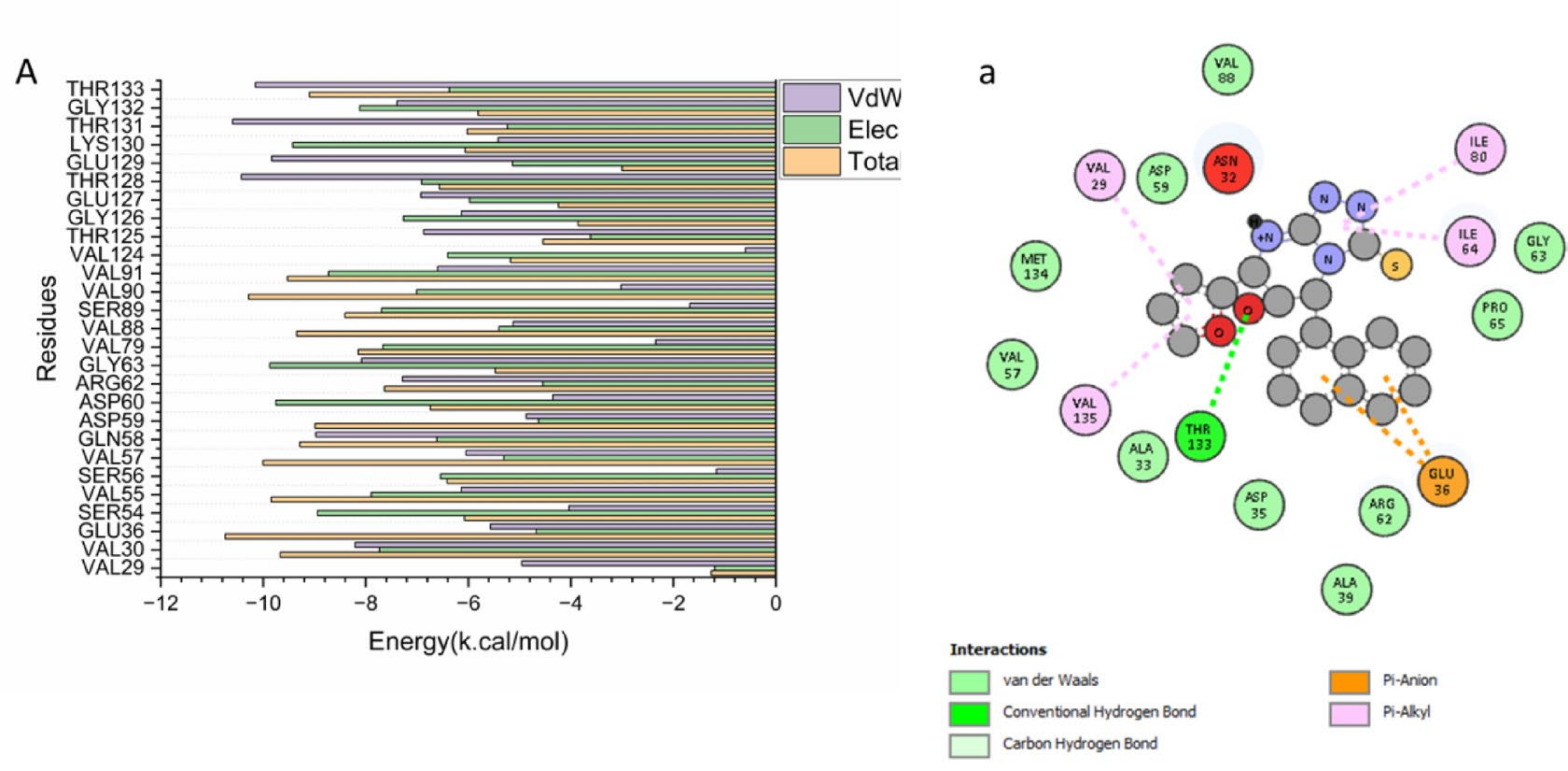
Per-residue decomposition plots showing the energy contributions to the binding and stabilization of compound 11 to the ATP binding site of DNA gyrase receptor.

As illustrated in Fig. 6A, the binding affinity of compound 11 is driven by favorable interactions with several residues. Dominant favorable contributors include Thr 128 (− 10.428 kcal/mol) and Thr 133 (− 10.153 kcal/mol). Notable stabilizing interactions also come from Gln 58 (− 8.976 kcal/mol), Gly 63 (− 8.081 kcal/mol), Val 30 (− 8.205 kcal/mol), and Arg 62 (− 7.282 kcal/mol), with additional support from Val 55 (− 6.137 kcal/mol) and Val 57 (− 6.045 kcal/mol).

### Ligand–residue interaction network profiles

A central goal in drug design is to modify molecular structures to optimize key properties, including enhanced bioavailability, reduced toxicity, and improved pharmacokinetic profiles^38^. The mechanism of action typically involves specific interactions between functional groups on the drug and complementary residues within the receptor’s active site, initiating a signal transduction pathway that culminates in the desired therapeutic effect^39^. Ligand-residue interaction profiling is a valuable method for decoding these binding events, clarifying the specific roles and interaction types of individual amino acids. Analysis reveals that hydrophobic contacts constitute the majority of interactions between compound 11 and the active site residues of the DNA gyrase receptor.

**Fig. 7:**
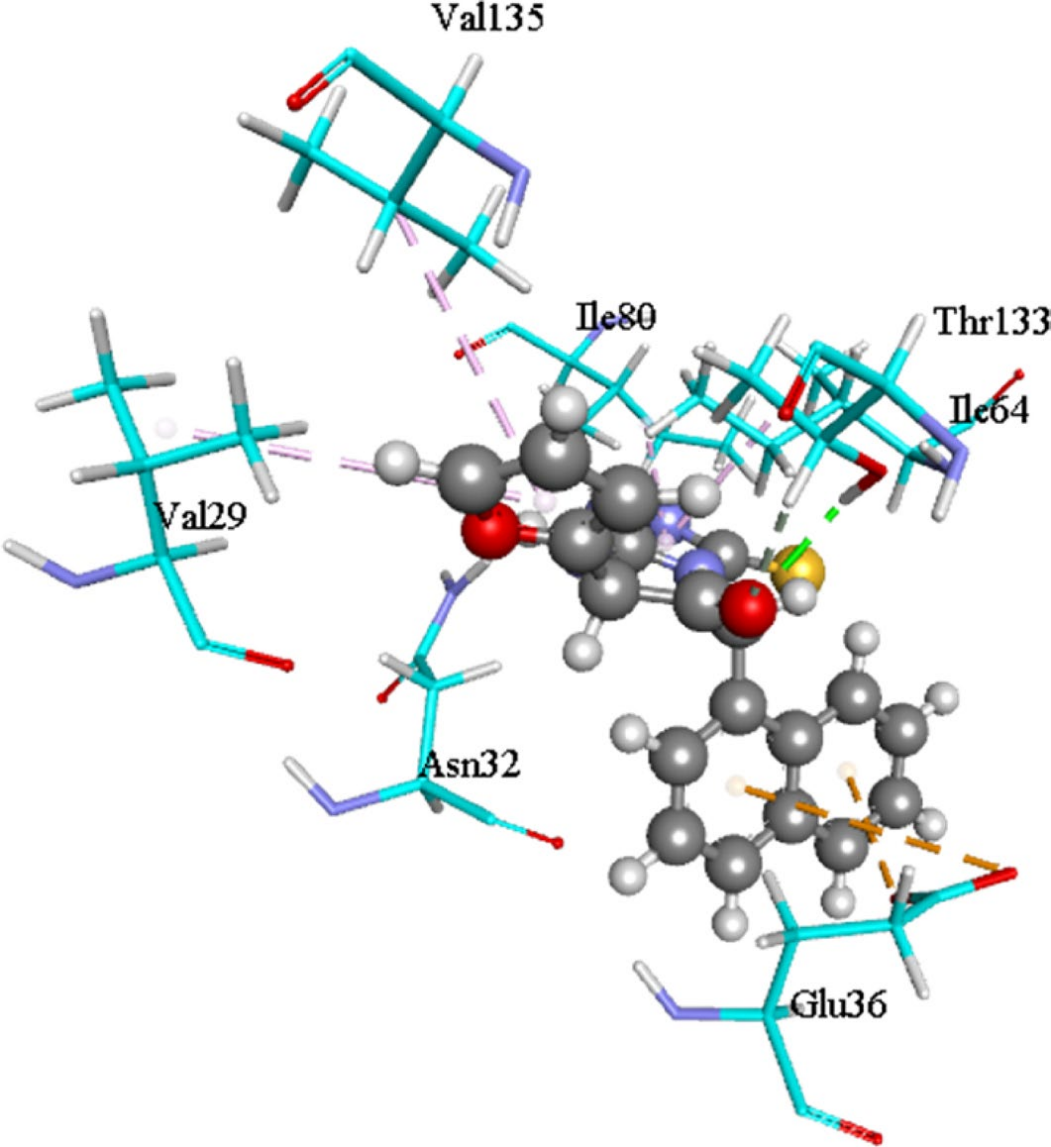
Molecular visualization of compound 11 at the binding site residue of DNA gyrase receptor.

As shown in Fig. 7, a stable hydrogen bond is formed between compound 11 and the ATP-binding residue Thr 133. Additional stabilization is provided by alkyl and/or apolar interactions with Ile 64, Ile 80, Val 29, and Val 135. Furthermore, a pi-anion interaction is observed between the ligand and Glu 36. This combination of polar and hydrophobic interactions defines the specific binding mode of compound 11 within the active site.

### Molecular docking and interaction analysis

The molecular docking and subsequent molecular dynamics (MD) simulations were performed to elucidate the binding mode and affinity of the synthesized compound 11 within the catalytic site of E. coli DNA gyrase (PDB ID: 6F86), a well-established target for antibacterial agents. The integration of docking poses with extensive 80 ns MD simulations and end-point binding free energy calculations via MM/GBSA provides a robust and dynamic perspective on the ligand-receptor complex stability and key interaction mechanisms.

The MM/GBSA binding free energy analysis revealed a strongly favorable total binding energy (ΔGbind = − 32.85 ± 0.26 kcal/mol) for compound 11. This profound affinity is primarily driven by dominant van der Waals contributions (ΔEvdW = − 46.96 ± 0.20 kcal/mol), underscoring exceptional shape complementarity and extensive hydrophobic packing within the ATP-binding pocket of DNA gyrase. The significant, yet comparatively smaller, electrostatic contribution (ΔEelec = − 7.95 ± 0.29 kcal/mol) suggests the presence of specific, stabilizing polar contacts. Although a substantial desolvation penalty (ΔGsolv =  + 22.06 ± 0.23 kcal/mol) was incurred—a common thermodynamic cost for displacing water molecules from a buried active site—the magnitude of the favorable intermolecular forces overwhelmingly compensates, confirming compound 11 as a potent putative inhibitor.

To decode the atomic determinants of this strong binding, a per-residue energy decomposition was conducted. The analysis identified several amino acids as critical anchors for ligand stabilization (Fig. 6). Residues Thr 128 (− 10.43 kcal/mol) and Thr 133 (− 10.15 kcal/mol) emerged as the most significant contributors, highlighting the importance of polar interactions, likely hydrogen bonds, with the hydroxyamino acid side chains. This observation aligns with recent studies on heterocyclic enzyme inhibitors, which emphasize that hydrogen bonding with conserved Thr/Ser residues in the active site is a cornerstone for high-affinity binding and inhibitory activity^40,41^. Substantial favorable contributions were also made by Gln 58, Gly 63, Val 30, and Arg 62, indicating a binding niche that exploits both polar (Gln, Arg) and aliphatic (Val) microenvironment features.

The ligand-residue interaction profile, visualized in Fig. 7, provides a detailed map of the specific contacts. A stable hydrogen bond was formed between a suitable donor/acceptor group on compound 11 and the backbone or sidechain of Thr 133, a residue frequently implicated in inhibitor binding within the ATPase domain. This key polar interaction is complemented by a network of extensive hydrophobic and alkyl contacts with residues Ile 64, Ile 80, Val 29, and Val 135, which encapsulate the ligand’s aromatic and aliphatic moieties. Such a hydrophobic cluster is critical for excluding water and enhancing binding affinity, a feature consistently reported in successful gyrase inhibitors and analogous systems^40,41^. Furthermore, a pi-anion interaction was identified between an electron-deficient aromatic ring of the ligand and the carboxylate group of Glu 36. This type of interaction is increasingly recognized as a valuable component of binding specificity and strength in medicinal chemistry, offering directional stability beyond typical hydrophobic effects^40^.

The consistency between our computational findings and the interaction patterns reported in recent, rigorous studies of enzyme inhibitors strengthens the validity of our proposed binding mode^40,41^. The synergy between a crucial hydrogen bond (Thr 133), a dense hydrophobic shell (Ile, Val), and a supportive electrostatic interaction (Glu 36) presents a compelling rationale for the observed high in vitro antimicrobial potency of compound 11. This integrated computational analysis not only validates the strong binding of compound 11 but also provides a structural blueprint for the rational design of next-generation pyrimidine-based antibiotics targeting DNA gyrase.

## Materials and methods

### Chemistry

All melting points were decided in open glass capillaries using an Electro thermal IA 9000 Series digital melting point apparatus (Electro thermal, Essex, UK) and are uncorrected. Elemental analyses were performed with all final compounds with an Elementary, Vario EL, Micro analytical Unit, Cairo University, Cairo Egypt, and were in good agreement (0.2%) with the calculated values. IR (KBr) spectra were recorded on a Shimadzu 408 spectrophotometer as a solid suspended in a potassium bromide disk. ^1^H and ^13^C NMR spectra were recorded on a Varian 500 MHZ”. “All chemical shifts were reported as d (ppm) scale using TMS as the standard, and coupling-constant values were given in Hz. The mass spectra (EI) were run at 70 eV with a Finnegan SSQ 7000 spectrometer (Thermo Instrument System Incorporated, USA), m/z values are indicated in Dalton.

### General procedures for the synthesis of (E)-1-(furan-2-yl)-3-(naphthalen-1-yl) prop 2-en-1-one (1)

A mixture of 2-acetylfuran (0.01 mol) and naphthalene-2-aldehydes (0.01 mol) in ethanol (30 ml) and 10% NaOH (15 ml) was added drop wise within 15 min. The reaction mixture was stirred for 3 h., and left overnight at 0ºC. The mixture was poured onto ice cold water and the obtained precipitate was filtered off and recrystallized from ethanol to give compound **1**.

Yield (80%); m.p. 102–103˚C; IR, ν, cm^−1^: 1666 (C=O); ^1^H-NMR (DMSO-*d*_*6*_, δ, ppm) 7.23–8.89 (m, 12H Ar–H); MS m/z (%): 248 (M^+^, 100%); Anal. calcd. For C_17_H_12_O_2_(248.28) (%): C, 82.24; H, 4.87; O, 12.89. Found (%): C, 82.28; H, 4.85; O, 12.87.

### General procedures for the synthesis of furan-2-yl (3-(naphthalen-1-yl) oxiran-2-yl) methanone (2)

Hydrogen peroxide (5 ml, 30%) was added portion wise to a mixture of compound **1** (0.01 mol) in acetone (30 ml) and methanol (15 ml) containing NaOH (1 g) at 0 °C with stirring. The reaction mixture was left overnight then cold water was added and the precipitated solid was filtered off, washed with cold water and crystallized from ethanol to give compound **2.**

Yield 67%, m.p. 132 °C; IR, ν, cm^−1^: 1672 (C=O); ^1^H-NMR (CDCl_3_, δ, ppm): 4.13–4.16 (d, J = 15 Hz, 1H, epoxy-H beta to C=O), 4.41–4.44 (d, J = 15 Hz, 1H, epoxy-H alpha to C=O), 7.25–8.54 (m, 10H, Ar–H); ^13^C-NMR (CDCl_3_-d6, δ, ppm) showed signals at: 60.12, 63.23, 125.21, 125.87, 126.41, 126.22, 127.19, 128.42, 129.26, 129.82, 130.11, 130.56, 130.91, 131.23, 131.68, 132.34, 195 (17 C); MS: m/z (%): 264 (M + , 40%); Anal. Calcd. for C_17_H_12_O_3_ (264.28) (%): C, 77.26; H, 4.58; O, 18.16 Found (%): C, 77.29; H, 4.57; O, 18.14.

### General procedures for the synthesis of 4-(furan-2-yl)-6-(naphthalen-1-yl)-2-thioxotetrahydropyrimidin5(2H)-one (3)

A mixture of compound **2** (0.01 mol) and (thiourea) (0.01 mol) in ethanolic potassium hydroxide (2 g in 100 ml ethanol) was refluxed for 4 h. The solvent was evaporated and the formed precipitate was washed several time with acidified cold water filtered off and recrystallized from dioxane solvent to give compound **3**.

Yield 76%; m.p. 232 °C; IR, ν, cm^−1^: 3130–3220(2NH), 1745(C=O), 1437(C=S); ^1^H-NMR (CDCl_3_-d6, δ, ppm): 3.47–3.50 (d, J = 15 Hz, 1H, Pyrimidine-H), 3.55–3.59(d, J = 15 Hz, 1H, Pyrimidine-H), 6.99–7.88 (m, 12 H, Ar–H + 2NH exchangeable by D_2_O). ^13^C-NMR (CDCl_3_-d6, δ, ppm) showed signals at: 45.15, 74.32, 128.24, 128.56, 128.91, 129.21, 129.53, 130.13, 130.37, 131.23, 131.41, 132.16, 132.53, 133.12, 133.43, 133.72, 178.16, 184.24 (18 C); MS: m/z (%): 322 (M + , 70%); Anal. Calcd. for C_18_H_14_N_2_O_2_S (322.08) (%): C, 67.06; H, 4.38; N, 8.69; O, 9.93; S, 9.94. Found (%): C, 67.09; H, 4.40; N, 8.67; O, 9.92; S, 9.92.

### General procedures for the synthesis of 7-(furan-2-yl)-5-(naphthalen-1-yl)-5H-thiazolo[3,2-a] pyrimidine-3,6(2H,7H)-dione (4)

A mixture of compound **3** (0.01 mol) with bromoacetic acid (0.01 mol) in acetic acid (30 ml) / acetic anhydride (15 ml) mixture in the presence of fused anhydrous sodium acetate (2 g) was refluxed for 15 h. The solution was cooled, gradually poured onto cold water and the formed precipitate was washed several times with water, filtered off and recrystallized from acetic acid to give compound **4**.

Yield 68%; m.p. 243 °C. IR, ν, cm^−1^: 1735, 1750 (2 C=O); ^1^H-NMR (CDCl_3_-d6, δ, ppm): 3.99–4.05(d, J = 10 Hz, 1H, thiazolo-H), 4.20–4.23 (d, J = 15 Hz, 1H, thiazolo-H), 3.93–3.95 (d, 1 H, J = 10 Hz, 1H pyrimidine proton), 3.95–3.97 (d, 1H, J = 10 Hz, 1H pyrimidine proton) and 7.15–7.89 (m, 10 H, Ar–H); MS: m/z (%): 362 (M + , 100%); Anal. Calcd. for C_20_H_14_N_2_O_3_S (362.06) (%): C, 66.28; H, 3.89; N, 7.73; O, 13.24; S, 8.85. Found (%): C, 66.30; H, 3.88; N, 7.74; O, 13.23; S, 8.84.

### General procedures for the synthesis of 7-(furan-2-yl)-2-methyl-5-(naphthalen-1-yl)-5H-thiazolo[3,2-a] pyrimidine-3,6(2H,7H)-dione (5)

A mixture of compound 3 (0.01 mol) with 2-bromopropionic acid (0.01 mol) in acetic acid (30 ml) / acetic anhydride (15 ml) mixture in the presence of fused anhydrous sodium acetate (2 g) was refluxed for 15 h.The solution was cooled, gradually poured onto cold water and the formed precipitate was washed several times with water, filtered off and recrystallized from acetic acid to give compound **5**.

Yield 63%; m.p. 253 °C; IR, ν, cm^−1^: 3495 (OH) and 1737 (2C=O); ^1^H NMR spectrum (CDCl_3_-d6, δ ppm): 4.01–4.06 (d, J = 15 Hz, 1H, Pyrimidine-H), 4.22–4.25 (d, J = 15 Hz, 1H, Pyrimidine-H), 2.76 (S, 3H, CH_3_), 8.39 (S, 1H, OH exchangeable by D_2_O), 7.29–7.90 (m, 10H, Ar–H); MS, m/z (%): 376 (M^+^, 81); Anal. Calcd. for C_21_H_16_N_2_O_3_S (376.43) (%): C, 67.00; H, 4.28; N, 7.44; O, 12.75; S, 8.52. Found (%): C, 67.03; H, 4.26; N, 7.46; O, 12.76; S, 8.51.

### General procedure for the synthesis of compounds (6a, 6b and 6c)

Formaldehyde (1 ml, 40%) was added to compound **3** (0.01 mol) in dry ethanol (30 ml) and the reaction mixture was heated for 5 min, cooled then 2nd amine (piperidine, morpholine and dimethyl amine) (0.01 mol) was added and the reaction mixture was stirred for overnight at room temperature. The formed solid was filtered off, dried and recrystallized from dioxane solvent to give compounds 6a, 6b, and 6c respectively.

### 4-(furan-2-yl)-6-(naphthalen-1-yl)-1-(piperidin-1-ylmethyl)-2-thioxotetrahydropyrimidin-5(2H)-one (6a)

From methanol; Yield 82%; m.p.237 °C; IR, ν, cm^−1^: 3295 (NH), 1719(C=O), 1429(C=S); ^1^H-NMR spectrum (DMSO-d6, δ, ppm): 1.18–1.50(m, 6H, piperidine-H), 2.31–2.53(m, 4H, piperidine-H), 3.55–3.58 (d, J = 15 Hz, 1H, pyrimidine-H), 3.72–3.75(d, J = 15 Hz, 1H, pyrimidine-H), 4.59–4.67 (d, 1H, N-CH_2_- N), 4.67–4.73(d,1H,N-CH_2_-N), 7.32–8.05(m, 11H, Ar–H + NH, D_2_O exchangeable); ^13^C-NMR (CDCl_3_-d6, δ, ppm): 44.32, 51.14, 53.18, 56.21, 60.28, 63.91, 65.13, 66.26, 70.36, 123.26, 124.28, 124.93, 125.46, 126.32, 126.81, 127.28, 129.18, 130.27, 131.28, 132.13, 132.74, 135.19, 176.32, 187.12 (24 C); MS: m/z (%): 419 (M^+^, 83); Anal. Calcd. for C_24_H_25_N_3_O_2_S (419.02) (%): C, 68.71; H, 6.01; N, 10.02; O, 7.63; S, 7.64. Found (%): C, 68.73; H, 6.03; N, 10.01; O, 7.61; S, 7.61.

### 4-(furan-2-yl)-1-(morpholinomethyl)-6-(naphthalen-1-yl)-2-thioxotetrahydropyrimidin-5(2H)-one (6b)

From methanol; Yield 82%; m.p.244 °C; IR, ν, cm^−1^: 3249 (NH), 1721 (C=O), 1430 (C=S); ^1^H-NMR (CDCl_3_, δ, ppm): 3.45–3.60 (m, 4H, NCH_2_), 3.75–4.09 (m, 4H, CH_2_O), 4.63–4.65(d, J = 15 Hz,1H Pyrimidine-H), 4.69–4.70(d, J = 15 Hz,1H, Pyrimidine-H), 5.33(S, 2H, N-CH_2_-N), 7.28–7.89(m, 10H, 10Ar-H) and (8.13, S, 1H, NH, D_2_O exchangeable); MS: m/z (%): 421 (M + , 75%), Anal. Calcd. for C_23_H_23_N_3_O_3_S (421.02) (%): C, 65.54; H, 5.50; N, 9.97; O, 11.39; S, 7.61 Found (%): C, 65.50; H, 5.51; N, 9.98; O, 11.38; S, 7.63.

### 1-((dimethylamino)methyl)-4-(furan-2-yl)-6-(naphthalen-1-yl)-2-thioxotetrahydropyrimidin-5(2H)-one (6c).

From methanol; Yield 85%; m.p.245 °C; IR, ν, cm^−1^: 3243 (NH), 1718 (C=O), 1418 (C=S); ^1^H-NMR (DMSO-d6, δ, ppm): 2.48(s,3H, CH_3_), 1.99 (s,3H, CH_3_), 4.28–4.30 (d, J = 15 Hz, 1H, Pyrimidine-H), 4.39–4.43(d, J = 15 Hz, 1H, Pyrimidine-H), 3.48 (dd, 2H, N-CH_2_-N), 7.29–7.57(m, 10H, Ar–H) and 11.21(S, 1H, NH, D_2_O exchangeable); MS: m/z (%): 379 (M + , 73%), Anal. Calcd. for C_21_H_21_N_3_O_2_S (379.5) (%): C, 66.47; H, 5.58; N, 11.07; O, 8.43; S, 8.45 Found (%): C, 66.49; H, 5.59; N, 11.02; O, 8.40; S, 8.43.

### General procedures for the synthesis of 4-(furan-2-yl)-2-hydrazineyl-6-(naphthalen-1-yl)-1,6-dihydropyrimidin-5(4H)-one (7)

A mixture of compound **3** (0.01 mol) and hydrazine hydrate (0.011 mol) in ethanol was refluxed for 7 h. The resulting solid was collected by filtration and recrystallized from ethanol to give compound **7**.

Yield (73%), m.p. 253 °C; IR, ν, cm^−1^: 3182–3382 (br 2NH, NH_2_), 1658 (C=O); ^1^H-NMR (DMSO-d6, δ, ppm): 3.32–3.34(d, J = 15 Hz, 1H, Pyrimidine-H), 3.36–3.38 (d, J = 15 Hz, 1H, Pyrimidine-H), 7.20–8.86(m, 11 H, Ar–H + NH exchangeable by D_2_O), 4.5 (S, 2H, NH_2_ exchangeable by D_2_O),10.12(S, 1H, NH exchangeable by D_2_O); MS: m/z (%): 320 (M + ,10%), Anal. Calcd. for C_18_H_16_N_4_O_2_ (320.4) (%): C, 67.49; H, 5.03; N, 17.49; O, 9.99 Found (%): C, 67.47; H, 5.05; N, 17.51; O, 9.97.

### General procedures for the synthesis of 5-(furan-2-yl)-7-(naphthalen-1-yl)-3,5-dihydrotetrazolo[1,5-a] pyrimidin-6(7H)-one (8)

To an ice-cold solution of compound **7** (0.01 mol) in glacial acetic (10 ml), a solution of sodium nitrite [prepared by dissolving sodium nitrite (0.01 mol) in water (3 ml) was added drowsily in an ice-bath. The reaction mixture was allowed to stand overnight at room temperature and then was poured into water. The formed solid was filtered of, washed with water, dried and recrystallized from dioxin to give **8**.

Yield (71%); m.p 233˚C; IR, ν, cm^−1^: 1730 (C=O) and 3338 (NH); ^1^H-NMR (CDCl_3_, δ, ppm): 3.83–3.85(d, J = 10 Hz, 1H, Pyrimidine-H), 3.73–3.75 (d, J = 10 Hz, 1H, Pyrimidine-H), 6.92–7.99(m, 10H, 10Ar-H) and 9.44(s, 1 H, NH exchangeable with D_2_O); MS: m/z (%):331(M + , 25%) Anal. Calcd. for C_18_H_13_N_5_O_2_ (320.1) (%): C, 65.25; H, 3.95; N, 21.14; O, 9.66. Found (%): C, 65.27; H, 3.97; N, 21.12; O, 9.64.

### General procedure for the synthesis of compounds 9a and 9b.

A solution of compound **7** (0.01 mol) in acetic acid and /or formic acid (20 ml) was refluxed for 7 h. The formed precipitate was filtered off and recrystallized from DMF to give compounds **9a**, and **9b** respectively.

### 7-(furan-2-yl)-3-methyl-5-(naphthalen-1-yl)-1,7-dihydro-[1,2,4]triazolo[4,3-a]pyrimidin-6(5H)-one (9a).

Yield (67%); m.p. 215˚C; IR, ν, cm^−1^: 1719 (CO) and 3255 (NH); ^1^H-NMR (DMSO-d6, δ, ppm): 3.13–3.15(d, J = 15 Hz, 1H, Pyrimidine-H), 3.29–3.30 (d, J = 15 Hz, 1H, Pyrimidine-H), 2.5(s, 3H, CH_3_), 7.25–8.11 (m, 10H, Ar H) and 9.94(s, 1H, NH, exchangeable by D_2_O); MS: m/z (%): 344(M + , 5%). Anal. Calcd. for C_20_H_16_N_4_O_2_ (344.4) (%): C, 69.76; H, 4.68; N, 16.27; O, 9.29 Found (%): C, 69.78; H, 4.69; N, 16.26; O, 9.27.

### 7-(furan-2-yl)-5-(naphthalen-1-yl)-1,7-dihydro-[1,2,4]triazolo[4,3-a]pyrimidin-6(5H)-one (9b)

Yield (58%); m.p. 220˚C; IR, ν, cm^−1^: 1722 (CO) and 3181 (NH); ^1^H-NMR (DMSO-d6, δ, ppm): 3.15–3.18(d, J = 15 Hz, 1H, Pyrimidine-H), 3.51–3.54(d, J = 15 Hz, 1H, Pyrimidine-H), 7.20–8.12 (m, 10H, Ar H) and 9.94(s, 1H, NH, exchangeable by D_2_O); MS: m/z (%):330 (M + , 7%). Anal. Calcd. for C_19_H_14_N_4_O_2_ (330.1) (%): C, 69.08; H, 4.27; N, 16.96; O, 9.69 Found (%): C, 69.06; H, 4.25; N, 16.97; O, 9.71.

### General procedure for the synthesis of 2-(3,5-dimethyl-1H-pyrazol-1-yl)-6-(furan-2-yl)-4-(naphthalen-1-yl)-1,6-dihydropyrimidin-5(4H)-one (10).

A mixture of compound **7** (0.01 mol) and acetyl acetone (0.03 mol) in absolute ethanol (20 ml) was refluxed for 12 h. The reaction mixture was concentrated, and the formed precipitate was filtered off and recrystallized from DMF to give compound **10.**

Yield (56%); m.p. 230˚C; IR, ν, cm^−1^: 3322 (NH) and 1754 (C=O). ^1^H-NMR ((DMSO-d6, δ, ppm): 2.47(S, 3H, CH_3_), 2.49(S, 3H, CH_3_), 3.35–3.37 (d, J = 10 Hz, 1H, Pyrimidine-H), 3.30–3.32(d, J = 10 Hz, 1H, Pyrimidine-H), 6.27(s, 1H, pyrazolo poroton), 7.17–7.94 (m, 10H, Ar H) and 10.34 (s, 1H, NH exchangeable by D_2_O); MS, m/z (%): 384 (M + , 85%). Anal. Calcd. for C_23_H_20_N_4_O_2_ (384.2) (%): C, 71.86; H, 5.24; N, 14.57; O, 8.32. Found (%): C, 71.89; H, 5.26; N, 14.54; O, 8.30.

### General procedure for the synthesis of 7-(furan-2-yl)-5-(naphthalen-1-yl)-3-thioxo-2,3,7,8-tetrahydro-[1,2,4]triazolo[4,3-a]pyrimidin-6(5H)-one (11).

To a warmed ethanolic potassium hydroxide solution [prepared by dissolving potassium hydroxide (0.01 mol) in ethanol (50 ml)], compound **8** (0.01 mol) and carbon disulphide (10 ml) were added. The mixture was heated on water bath at 80 °C under reflux for 10 h, and then it was poured into water, neutralized by diluted hydrochloric acid. The formed solid was collected and recrystallized from ethanol to give **11.**

Yield (77%); m. p. 195 °C. IR, ν, cm^−1^: 1740 (C=O), 3129(NH), 1434(C=S), 2929(C=N); ^1^H-NMR (CDCl_3_-d6, δ, ppm): 3.28–3.32 (d, J = 15 Hz, 1H, pyrimidine-H), 3.56–3.59 (d,J = 15,1H,pyramidine-H),7.13–7.95(m,10H,11Ar-H) and 10.20, 10.95 (2H, 2NH, D_2_O exchangeable); MS: m/z (%): 362. Anal. Calcd. for C_19_H_14_N_4_O_2_S (362.4) (%): C, 62.97; H, 3.89; N, 15.46; O, 8.83; S, 8.85. Found (%):C, 62.95; H, 3.87; N, 15.47; O, 8.84; S, 8.87.

### Antioxidant activity

The free radical-scavenging properties of compounds were investigated using DPPH^41^. One ml of the compounds was mixed with one ml of a 0.3 mM DPPH methanolic solution. After shaking, the mixture was kept at room temperature (30 °C) in a dark box for half an hour. At 517 nm, the resultant solution’s absorbance was measured.

The following equation was used to determine the inhibitory percentage of DPPH:

$${\mathrm{Scavenging}}\;{\mathrm{activity}}\;\left( \% \right) = \left[ {\left( {\frac{{{\text{Control }}\;{\text{absorbance }} - {\text{ sample }}\;{\mathrm{absorbance}}){ }}}{{{\text{absorbance }}\;{\mathrm{of}}\;{\mathrm{control}}}}} \right) \times 100\% } \right]$$

### Antimicrobial activity

#### Microorganisms

For powder samples, the in vitro antibacterial activity was evaluated. Gram-positive bacterial strains (*B. subtilis ATCC-6633*), gram-negative bacterial strains (*E. coli ATCC-25922*), and fungal species (*A. niger NRRL-3* and *C. albicans ATCC-10231*) were among the microorganisms used in this investigation. The American Type Culture Collection (ATCC, Rockville, MD, USA) and the Northern Utilization Research and Development Division, US Department of Agriculture, Peoria, Illinois, USA (NRRL) provided the microorganisms. The bacterial strains were subcultured in fresh nutritional agar (NA) medium (Merck, Darmstadt, Germany) for a full day prior to the test in order to revive them for bioassay. Before the studies were conducted, the fungi were cultivated on potato dextrose agar (PDA) (Lab M., Bury, Lancashire, UK) for 7 days at 28 °C.

#### Inoculum preparation

On slopes of nutritional agar and potato dextrose agar, stock cultures were kept at 4 °C. A lapful of cells from the stock cultures were transferred to test tubes containing Mueller–Hinton broth (MHB) (Lab M Limited, Bury, Lancashire, UK) for bacteria and Sabouraud dextrose broth (SDB) (Lab M., Bury, Lancashire, UK) for fungi in order to create active cultures for the tests. The cultures were incubated at 37 °C and 25 °C, respectively, for 24 h without stirring. 0.2 ml of culture was infected with 5 ml of MHB and SDB, and it was incubated until the turbidity was equal to that of the standard 0.5 McFarland solution at 625 nm (A = 0.08 to 0.1), or 1.5 × 108 cfu/ml.

#### In vitro bioassay method of antimicrobial activities

The agar diffusion experiment, as outlined by Perez et al.^39^, was used to assess the powder samples’ in vitro antibacterial activity.

Sterile Petri dishes previously labeled with the test bacteria were filled with a 0.1 ml aliquot of an 18-h broth culture of the aforementioned bacteria, adjusted to the turbidity equivalent of 0.5 McFarland standards^42,43^. To ensure that the bacteria were evenly dispersed throughout the medium, the plates were aseptically filled with molten, sterile Muller-Hinton agar and gently rotated. The agar plates were left to harden. Using Mueller–Hinton agar (Lab M Limited, Bury, Lancashire, UK) and the agar well diffusion method outlined by Jorgensen and Turnidge^44^, the antibacterial screening bioassay was carried out. The plates were left to diffuse for two hours at 4 °C. Three duplicates of the experiment were carried out. For bacterial strains, all plates were incubated for 24 h at 37 °C, and for fungal strains, for 48 h at 28–30 °C. Millimeters were used to measure and label the clearance zones surrounding the wells^45^. Fungal antibiotics (clotrimazole 50 µg and cycloheximide 50 µg) and standard antibiotics (tetracycline 30 µg and novobiocin 30 µg) were used as positive controls for bacteria and fungi, respectively.

### System preparation and molecular docking

The crystal structure of *E. coli* DNA gyrase was obtained from the Protein Data Bank (PDB ID: 6F86)^46^. Initial preparation of the protein structure was performed with UCSF Chimera software^47^. The protonation states of ionizable residues were adjusted and optimized for a physiological pH of 7.5 using the PROPKA algorithm^48^.

Separately, the two-dimensional (2D) structure of the synthesized ligand was constructed in ChemBioDraw Ultra 12.1^49^. This structure was then imported into Avogadro software^50^ for geometry optimization and energy minimization, which was carried out using the MMFF94 force field and the steepest descent algorithm.

In the final preparation step for molecular dynamic, all hydrogen atoms were systematically removed from both the protein and ligand structures using UCSF Chimera^47^.

### Molecular docking

Molecular docking calculations were carried out using AutoDock Vina^51^, with Gasteiger partial charges applied to the structures^52^. The graphical interface provided by MGL Tools was used to define the necessary AutoDock atom types^53^.

For the docking simulation, a grid box was centered at coordinates x = − 62.27, y = 30.74, z = 61.12. The dimensions of the search space were set to 20 Å in each direction, and the exhaustiveness parameter was defined as 8. The Lamarckian genetic algorithm^54^ was employed to generate the ligand poses, which were subsequently ranked in order of increasing binding affinity (i.e., declining docking energy).

### Molecular dynamic (MD) simulations

Molecular dynamics (MD) simulations provide a powerful tool for investigating the physical movements of atoms and molecules within biological systems, offering insights that are difficult to obtain through other experimental means^55^. This approach yields a detailed perspective on dynamic processes such as conformational changes and molecular interactions^55^. In this study, all simulations were performed using the GPU-accelerated PMEMD engine within the AMBER 18 software suite^56^.

The partial atomic charges for each ligand were derived using the General Amber Force Field (GAFF) via the ANTECHAMBER utility^57^. Each system was prepared using the LEaP module in AMBER 18, where it was explicitly solvated in an orthorhombic box of TIP3P water molecules, maintaining a 10 Å buffer between the solute and the box edges. System neutrality was achieved by adding Na⁺ and Cl⁻ counterions. Energy minimization was conducted in two stages: first, a 2000-step minimization with a 500 kcal/mol/Å^2^ restraint on solute atoms, followed by a 1000-step unrestrained minimization using the conjugate gradient algorithm.

Prior to production, each system was gradually heated from 0 to 300 K over 500 ps in the NVT ensemble, maintaining consistent atom counts and volume. During heating, a harmonic restraint of 10 kcal/mol/Å^2^ was applied to the solute, with a collision frequency of 1 ps^−1^. This was followed by a 500 ps equilibration period at 300 K under constant pressure (NPT ensemble) conditions.

Production MD simulations were performed in the isothermal-isobaric (NPT) ensemble at 300 K and 1 bar, maintained using the Berendsen barostat with a pressure coupling constant of 2 ps. Temperature was regulated with a Langevin thermostat (collision frequency of 1 ps⁻^1^). The SHAKE algorithm was employed to constrain bonds involving hydrogen atoms. All integrations used a 2 fs timestep with the SPFP precision model, and simulations were initiated with randomized atomic velocities.

### Post-MD analysis

After saving the trajectories obtained by MD simulations every 1 ps, the trajectories were analyzed using the AMBER18 suite’s CPPTRAJ module. The Origin data analysis program and Chimera were used to create all graphs and visualizations^57^.

### Thermodynamic calculation

The Poisson-Boltzmann or generalized Born and surface area continuum solvation (MM/PBSA and MM/GBSA) approach has been found to be useful in the estimation of ligand-binding affinities^58^. The Protein–Ligand complex molecular simulations used by MM/GBSA and MM/PBSA compute rigorous statistical-mechanical binding free energy within a defined force field.

Binding free energy averaged over 200 snapshots extracted from the entire 20 ns trajectory. The estimation of the change in binding free energy (ΔG) for each molecular species (complex, ligand, and receptor) can be represented as follows:

1 $$\Delta {\mathrm{G}}_{{{\mathrm{bind}}}} = {\mathrm{G}}_{{{\mathrm{complex}}}} - {\mathrm{G}}_{{{\mathrm{receptor}}}} - {\mathrm{G}}_{{{\mathrm{ligand}}}}$$

2 $$\Delta {\mathrm{G}}_{{{\mathrm{bind}}}} = {\mathrm{E}}_{{{\mathrm{gas}}}} + {\mathrm{G}}_{{{\mathrm{sol}}}} - {\text{TS }}$$

3 $${\mathrm{E}}_{{{\mathrm{gas}}}} = {\mathrm{E}}_{{{\mathrm{int}}}} + {\mathrm{E}}_{{{\mathrm{vdw}}}} + {\mathrm{E}}_{{{\mathrm{ele}}}} { }$$

4 $${\mathrm{G}}_{{{\mathrm{sol}}}} = {\mathrm{G}}_{{{\mathrm{GB}}}} + {\mathrm{G}}_{{{\mathrm{SA}}}}$$

5 $${\mathrm{G}}_{{{\mathrm{SA}}}} = \gamma {\text{SASA }}$$

The terms Egas, Eint, Eele, and Evdw symbolize the gas-phase energy, internal energy, Coulomb energy, and van der Waals energy. The Egas was directly assessed from the FF14SB force field terms. Solvation-free energy (Gsol) was evaluated from the energy involvement from the polar states (GGB) and non-polar states (G). The non-polar solvation free energy (GSA) was determined from the Solvent Accessible Surface Area (SASA)(21,22) using a water probe radius of 1.4 Å. In contrast, solving the GB equation assessed the polar solvation (GGB) contribution. Items S and T symbolize the total entropy of the solute and temperature, respectively. The MM/GBSA-binding free energy method in Amber18 was used to calculate the contribution of each residue to the total binding free energy.

## Conclusion

Chalcone **1** was produced and reacting with hydrogen peroxide afforded oxiran derivatives **2**, which reacted with thiourea to give pyrimidine derivatives **3** as starting material. Reaction of pyrimidine derivatives **3** with bromoacetic acid and/or 2-bromopropionic acid respectively, afforded thiazolopyrimidine derivatives **4** and **5**. Reaction of pyrimidine derivatives **3** with formaldehyde and secondary amine namely, piperidine, morpholine and/or dimethyl amine respectively, afforded Mannich bases **6a-c**. Treatment of compound **3** with hydrazine hydrate to afford the corresponding hydrazinopyrimidine derivatives **7**. Diazotization of compound **7** in presence of glacial acetic and sodium nitrite afforded tetrazolopyrimidine derivatives **8**. Also, compound **7** reacted with acetic acid and /or formic acid, respectively to afford triazolopyrimidine derivatives **9a, b** respectively. Further condensation of compound **7** with acetyl acetone in absolute ethanol gave diazolopyrimidine derivatives **10**. Finally, compound 7 reacted with carbon disulphide in ethanolic potassium hydroxide solution to afford triazolopyrimidine derivatives **11**. Their pharmacological potential was evaluated through integrated in vitro and in silico approaches. The experimental biological screening revealed that several compounds, particularly **5, 9a, and 11**, exhibited significant dual antioxidant and broad-spectrum antimicrobial activities against both bacterial (*E. coli*, *B. subtilis*) and fungal (*A. niger*, *C. albicans*) strains. Notably, compound **11** emerged as the most potent candidate, showing inhibitory zones comparable to the standard drugs streptomycin and cycloheximide. Critically, these experimental findings are strongly corroborated and explained by the computational analyses**.** Molecular docking and subsequent molecular dynamics simulations demonstrated that compound **11** forms a highly stable complex with the ATP-binding site of *E. coli* DNA gyrase, a key antibacterial target. The MM/GBSA binding free energy calculation yielded a strongly favorable ΔGbind of − 32.85 kcal/mol, primarily driven by extensive van der Waals interactions and a key hydrogen bond with Thr 133. The stability of this complex was confirmed by MD simulations, which showed that compound **11** reduced protein flexibility and increased structural compactness—dynamic signatures of a tight-binding inhibitor. Therefore, the exceptional in vitro antimicrobial potency of compound 11 can be directly attributed to its optimized molecular interactions and stable binding mode within the DNA gyrase active site**,** as predicted by the in silico studies. This compelling convergence of experimental and computational evidence validates our integrated design and screening strategy. In summary, compound **11** is identified as a highly promising lead molecule, combining potent observed bioactivity with a computationally validated mechanism of action. This work underscores the value of combining synthesis, biological evaluation, and computational modeling in the rational development of new antimicrobial agents.

## Supplementary Information

Below is the link to the electronic supplementary material.

Supplementary Material 1

## Data Availability

All data generated or analyzed are included in this published article and its supplementary information file.

## References

[1] Hassan, S. A. et al. Synthesis and pharmacological profiling of mono imine Schiff bases: Dual anti-inflammatory and antibacterial activity with computational insights. *J. Mol. Struct.* **1351**, 144096. 10.1016/j.molstruc.2025.144096 (2026).

[2] Hassan, S. A. et al. Synthesis and characterization of azo-azomethine derivatives bearing thiazole moiety: In vitro antimicrobial, in vitro and in vivo anti-inflammatory, and cytotoxicity assessment, accompanied by computational molecular docking, RDG, ELF, DFT, and MEP analysis. *J. Mol. Struct.* **1318**, 139294. 10.1016/j.molstruc.2024.139294 (2024).

[3] Hassan, S. A. et al. Synthesis of new 4-thiazolidinone bearing thiazole, assess anticancer, and antimicrobial activities: Insights from DFT, and molecular docking. *J. Mol. Struct.* **1346**, 143181. 10.1016/j.molstruc.2025.143181 (2025).

[4] Khalaf, H. S. et al. Synthesis, molecular docking, and pharmacological evaluations of novel pyrimidine derivatives. *Chem. Biodivers.* **22**, e202500477. 10.1002/cbdv.202500477 (2025). 40192213 10.1002/cbdv.202500477

[5] Rani, P. et al. Electro-organic synthesis of C-5 sulfenylated amino uracils: Optimization and exploring Topoisomerase-I based anti-cancer profile. *Bioorg. Chem.* **138**, 106660. 10.1016/j.bioorg.2023.106660 (2023). 37320914 10.1016/j.bioorg.2023.106660

[6] Khalaf, H. S. et al. Reactivity of 2-((3-cyano-4-(4-fluorophenyl)-6-(naphthalen-2-yl) pyridin-2-yl) oxy) acetohydrazide toward some reagents for preparing a promising anticancer agents and molecular docking study. *Chem. Biodivers.* **22**, e202403463. 10.1002/cbdv.202403463 (2025). 39910835 10.1002/cbdv.202403463

[7] Tangeda, S. J. & Garlapati, A. Synthesis of new pyrrolo [2,3d] pyrimidine derivatives and evaluation of their activities against human colon cancer cell lines. *Eur. J. Med. Chem.* **45**, 1453–1458. 10.1016/j.ejmech.2009.12.050 (2010). 20163895 10.1016/j.ejmech.2009.12.050

[8] Rahaman, Sk. A. et al. Synthesis and anti-histaminic activity of some novel pyrimidines. *Saudi Pharm. J.* **17**, 255–258. 10.1016/j.jsps.2009.08.001 (2009). 23964169 10.1016/j.jsps.2009.08.001 PMC3731017

[9] Abdelkhalek, A. S. et al. Synthesis new multitarget-directed ligands having thienopyrimidine nucleus for inhibition of 15-lipoxygenases, and pro-inflammatory cytokines. *Eur. J. Med. Chem.* **256**, 115443. 10.1016/j.ejmech.2023.115443 (2023). 37182334 10.1016/j.ejmech.2023.115443 PMC10247423

[10] Manzoor, S. et al. Synthesis and pharmacological evaluation of novel triazole-pyrimidine hybrids as potential neuroprotective and anti-neuro inflammatory agents. *Pharm. Res.* **40**(1), 167–185. 10.1007/s11095-022-03429-1 (2023). 36376607 10.1007/s11095-022-03429-1 PMC10964282

[11] Abd El-Gwaad, A. A. et al. Pharmacological evaluation as analgesic and anti-inflammatory and molecular docking of newly synthesized nitrogen heterocyclic derivatives. *Sci. Rep.* **15**, 44309. 10.1038/s41598-025-31238-0 (2025). 41422127 10.1038/s41598-025-31238-0 PMC12722756

[12] Hassan, S. A. & Abdullah, M. N. Synthesis of new imidazole derivatives bearing a morpholine moiety: Molecular docking, DFT (FOMs, MEP, RDG, ELF, LOL) analysis, and evaluation of antimicrobial, anti-inflammatory (in vivo/in vitro), toxicity, and hemolytic activities. *J. Mol. Struct.* **1347**, 143286. 10.1016/j.molstruc.2025.143286 (2025).

[13] Hamdy, N. A. et al. Discovery of novel tetralin-thiazole conjugates as VEGFR-2 inhibitors with anticancer potential: An integrated computational and biological study. *J. Mol. Struct.* **1349**, 143602. 10.1016/j.molstruc.2025.143602 (2026).

[14] Adel, D. et al. Pyrazolo[3,4-d]pyrimidine derivatives as EGFRT790M and VEGFR-2 dual TK inhibitors: Design, synthesis, molecular docking, ADMET profile and anticancer evaluations. *J. Mol. Struct.* **1291**, 136047. 10.1016/j.molstruc.2023.136047 (2023).

[15] Al-Otaibi, J. S. et al. Insights into solvation, chemical reactivity, structural, vibrational and anti-hypertensive properties of a thiazolopyrimidine derivative by DFT and MD simulations. *Struct. Chem.* **33**, 1271–1283. 10.1007/s11224-022-01931-1 (2022).

[16] Irshad, N. et al. Antihypertensive potential of selected pyrimidine derivatives: Explanation of underlying mechanistic pathways. *Biomed. Pharmacother.* **139**, 111567. 10.1016/j.biopha.2021.111567 (2021). 33848773 10.1016/j.biopha.2021.111567

[17] Geist, J. G. et al. Thiazolopyrimidine inhibitors of 2- methylerythritol 2, 4-cyclodiphosphate synthase (IspF) from *Mycobacterium tuberculosis* and *Plasmodium falciparum*. *Chem. Med. Chem.* **5**, 1092–1101. 10.1002/cmdc.2010000832010 (2010). 20480490 10.1002/cmdc.201000083

[18] El-Sayed, A. M. et al. Synthesis and antimicrobial activity of newly synthesized 4-substituted-pyrazolo [3, 4- d] pyrimidine derivatives. *Med. Chem. Res.* **26**, 1107–1116. 10.1007/s00044-017-1814-0 (2017).

[19] Sanad, S. M. & Mekky, A. E. Three-component regioselective synthesis and antibacterial evaluation of new arene-linked bis (pyrazolo [1, 5-a] pyrimidine) hybrids. *Synth. Commun.* **53**(9), 658–672. 10.1080/00397911.2023.2191854 (2023).

[20] Kumar, D. et al. 4-Aminoquinoline-pyrimidine hybrids: Synthesis, antimalarial activity, heme binding and docking studies. *Eur. J. Med. Chem.* **89**, 490–502. 10.1016/j.ejmech.2014.10.061 (2015). 25462261 10.1016/j.ejmech.2014.10.061

[21] Suresh, L. et al. An expeditious four-component domino protocol for the synthesis of novel thiazolo [3, 2-a] thiochromeno [4, 3-d] pyrimidine derivatives as antibacterial and antibiofilm agents. *Bioorg. Med. Chem.* **24**, 3808–3817. 10.1016/j.bmc.2016.06.025 (2016). 27344213 10.1016/j.bmc.2016.06.025

[22] Ishwar Bhat, K. et al. Synthesis, pharmacological and biological screening of some novel pyrimidine derivatives. *Med. Chem. Res.* **23**, 3458–3467. 10.5958/0974-360X.2017.00190.1 (2014).

[23] Sriharsha, S. N. et al. Novel β-L-1, 3-thiazolidine pyrimidine nucleoside analogues: Design, synthesis, molecular docking, and anti-HIV activity. *J. Mol. Struct.* **1294**, 136304. 10.1016/j.molstruc.2023.136304 (2023).

[24] Dansena, H. et al. Pharmacological potentials of pyrimidine derivative: A review. *Asian J. Pharm. Clin. Res.* **8**, 171–177 (2015).

[25] Raval, K. & Ganatra, T. Basics, types and applications of molecular docking: A review. *IP Int. J. Compr. Adv. Pharm.* **7**, 12–16. 10.18231/j.ijcaap.2022.003 (2022).

[26] Khalaf, H. S. et al. Synthesis, biological evaluation, and molecular docking studies of indole-based heterocyclic scaffolds as potential antibacterial agents. *Chem. Biodivers.* **22**, e202402325. 10.1002/cbdv.202402325 (2024). 39433506 10.1002/cbdv.202402325

[27] Sroor, F. M. et al. Design, synthesis, structure elucidation, antimicrobial, molecular docking, and SAR studies of novel urea derivatives bearing tricyclic aromatic hydrocarbon rings. *Arch. Pharm.* **357**, 2300738. 10.1002/ardp.202300738 (2024). 10.1002/ardp.202300738 38466125

[28] Khalaf, H. S. et al. Enhanced antibacterial and antioxidant capabilities using indole-modified 1-phenyl-1H-pyrazolo[3,4-b] pyridine-5-carbonitrile derivatives, molecular docking evaluation and in silico ADMET prediction. *RSC Adv.* **15**, 47255. 10.1039/D5RA07372C (2025). 41333651 10.1039/d5ra07372c PMC12668186

[29] Hasanin, M. et al. Synthesis of novel heterocyclic compounds based on dialdehyde cellulose: Characterization, antimicrobial, antitumor activity, molecular dynamics simulation and target identification. *Cellulose* **1**, 8355–8374. 10.1007/s10570-021-04063-7 (2021).

[30] Machaba, K. E. et al. Induced mutation proves a potential target for TB therapy: A molecular dynamics study on LprG. *Cell. Biochem. Biophys.* **76**, 345–356. 10.1007/s12013-018-0852-7 (2018). 30073572 10.1007/s12013-018-0852-7

[31] Pan, L. et al. Molecular dynamics study of Zn(Aβ) and Zn(Aβ)2. *PLoS ONE* **8**(9), 70681–70688. 10.1371/journal.pone.0070681 (2013). 10.1371/journal.pone.0070681 PMC3785486 24086248

[32] Wijffels, G. et al. Conservation of eubacterial replicases. *IUBMB Life* **57**(6), 413–419. 10.1080/15216540500138246 (2005). 16012050 10.1080/15216540500138246

[33] Khalaf, H. S. et al. Synthesis, characterization and frequency-dependent dielectric applications of new indole–oxindole-based on propanenitrile derivatives. *Egypt. J. Chem.* **69**, 1–7. 10.21608/ejchem.2025.427898.12400 (2026).

[34] Cournia, Z. et al. Relative binding free energy calculations in drug discovery: Recent advances and practical considerations. *J. Chem. Inf. Model.* **57**, 2911–2937. 10.1021/acs.jcim.7b00564 (2017). 29243483 10.1021/acs.jcim.7b00564

[35] Ejalonibu, M. A. et al. Dual targeting approach for *Mycobacterium tuberculosis* drug discovery: Insights from DFT calculations and molecular dynamics simulations. *Struct. Chem.* **31**, 1–15. 10.1007/s11224-019-01422-w (2019).

[36] Khalaf, H. S. et al. Reactivity of (3-(4-fluorophenyl)oxyran-2-yl)(naphthalene-2yl)methanone toward some nucleophiles for preparing promising agents with antimicrobial and antioxidant activities. *Chem. Sel.* **9**, e202404371. 10.1002/10.1002/slct.202404371 (2024).

[37] Mansoor, S. et al. Green synthesis of a MnO-GO-Ag nanocomposite using leaf extract of *Fagonia arabica* and its antioxidant and anti-inflammatory performance. *Nano Struct. Nano Objects* **29**, 100835. 10.1016/j.nanoso.2021.100835 (2022).

[38] Aziz, D. M. et al. A synergistic investigation of azo-thiazole derivatives incorporating thiazole moieties: A comprehensive exploration of their synthesis, characterization, computational insights, solvatochromism, and multimodal biological activity assessment. *RSC Adv.* **13**, 34534. 10.1039/d3ra06469g (2023). 38024963 10.1039/d3ra06469g PMC10668576

[39] Zamora, L. & Perez-Gracia, M. Using digital photography to implement the McFarland method. *J. R. Soc. Interface* **9**, 1892–1897. 10.1098/rsif.2011.0809 (2012). 22337631 10.1098/rsif.2011.0809 PMC3385748

[40] Jorgensen, J. H. & Turnidge, J. D. Susceptibility test methods: Dilution and disk diffusion methods. *Man. Clin. Microbiol.*10.1128/9781555817381.ch71 (2015).

[41] Abd El-Gwaad, A. A. et al. Novel Triazolopyridine derivatives: Synthesis, antimicrobial, anticancer evaluation and molecular docking studies. *J. Biochem. Mol. Toxicol.* **40**, e70743. 10.1002/jbt.70743 (2026). 41674302 10.1002/jbt.70743

[42] Narramore, S. et al. New insights into the binding mode of pyridine-3-carboxamide inhibitors of E. coli DNA gyrase. *Bioorg. Med. Chem.* **27**(16), 3546–3550 (2022). 10.1016/j.bmc.2019.06.015 31257079

[43] Hassan, S. A. et al. Design, synthesis, and computational analysis (molecular docking,DFT, MEP, RDG, ELF) of diazepine and oxazepine sulfonamides: Biological evaluation for in vitro and in vivo anti‑inflammatory, antimicrobial, and cytotoxicity predictions. *Mol. Diversity* **29**, 2367–2389. 10.1007/s11030-024-10996-5 (2025). 10.1007/s11030-024-10996-5 39356365

[44] Li, H. et al. Very fast empirical prediction and rationalization of protein pKa values. *Proteins* **61**(4), 704–21. Available from: https://pubmed.ncbi.nlm.nih.gov/16231289/ (2022). 10.1002/prot.20660 16231289

[45] Khalaf, H. S. et al. Synthesis, molecular docking, ADMET studies and biological evaluation of fused pyrazolopyridopyrimidine derivatives as antioxidant and antimicrobial agents. *Sci. Rep.*10.1038/s41598-025-30217-9 (2025). 41381722 10.1038/s41598-025-30217-9 PMC12701012

[46] Hassan, S. A. et al. Design and synthesis of oxazepine derivatives from sulfonamide Schiff bases as antimicrobial and antioxidant agents with low cytotoxicity and hemolytic prospective. *J. Mol. Struct.* **1292**, 136121. 10.1016/j.molstruc.2023.136121 (2023).

[47] Trott, O. et al. Improving the speed and accuracy of docking with a new scoring function, efficient optimization, and multithreading. *J. Comput. Chem.* **31**(2), 455–461. 10.1002/jcc.21334 (2010). 19499576 10.1002/jcc.21334 PMC3041641

[48] Khalaf, H. S. et al. Synthesis, docking, computational studies, and antimicrobial evaluations of new dipeptide derivatives based on nicotinoyl-glycylglycine hydrazide. *Molecules* **25**, 3589. 10.3390/molecules25163589 (2020). 32784576 10.3390/molecules25163589 PMC7464391

[49] Aziz, D. M. et al. New azo-azomethine derivatives: Synthesis, characterization, computational, solvatochromic UV–Vis absorption and antibacterial studies. *J. Mol. Struct.* **1284**, 135451. 10.1016/j.molstruc.2023.135451 (2023).

[50] Morris, G. M. et al. Automated docking using a Lamarckian genetic algorithm and an empirical binding free energy function. *J. Comput. Chem.* **19**(14), 1639–1662. Available from: https://onlinelibrary.wiley.com/terms-and-conditions (1998).

[51] Hospital, A. et al. Molecular dynamics simulations: Advances and applications. *Adv. Appl. Bioinform. Chem.* **8**, 37–47. 10.2147/AABC.S70333 (2015). 26604800 10.2147/AABC.S70333 PMC4655909

[52] Lee, T. S. et al. GPU-accelerated molecular dynamics and free energy methods in Amber18: Performance enhancements and new features. *J. Chem. Inf. Model.* **22**, 2043–2050. 10.1021/acs.jcim.8b00462 (2018). 10.1021/acs.jcim.8b00462 PMC6226240 30199633

[53] Aziz, D. M. et al. Azo-azomethine complex activity and sensor potential toward Ca(II) ion in life samples: The spectroscopic and morphological studies. *J. Mol. Struct.* **1293**, 136204. 10.1016/j.molstruc.2023.136204 (2023).

[54] Roe, D. R. & Cheatham, T. E. PTRAJ and CPPTRAJ: Software for processing and analysis of molecular dynamics trajectory data. *J. Chem. Theory Comput.* **9**(7), 3084–3095. 10.1021/ct400341p (2013). 26583988 10.1021/ct400341p

[55] Ylilauri, M. & Pentikäinen, O. T. MMGBSA as a tool to understand the binding affinities of filamin-peptide interactions. *J. Chem. Inf. Model.* **53**, 2626–2633. 10.1021/ci4002475 (2013). 23988151 10.1021/ci4002475

[56] Greenidge, P. A., Kramer, C., Mozziconacci, J. C. & Wolf, R. M. MM/GBSA binding energy prediction on the PDBbind data set: Successes, failures, and directions for further improvement. *J. Chem. Inf. Model.* **53**, 201–209. 10.1021/ci300425v (2013). 23268595 10.1021/ci300425v

[57] Seifert, E. OriginPro 9.1: Scientific data analysis and graphing software—Software review. *J. Chem. Inf. Model.* **54**, 1552. 10.1021/ci500161d (2014). 24702057 10.1021/ci500161d

[58] Hou, T., Wang, J., Li, Y. & Wang, W. Assessing the performance of the MM/PBSA and MM/GBSA methods. 1. The accuracy of binding free energy calculations based on molecular dynamics simulations. *J. Chem. Inf. Model.* **51**, 69–82. 10.1021/ci100275a (2011). 21117705 10.1021/ci100275a PMC3029230

