# Supplementary material for: Synthesis, antioxidant and antimicrobial activities, molecular docking study of new pyrimidine derivatives
Source: Sci Rep. 2026 Apr 13;16:12354. doi: 10.1038/s41598-026-45654-3 (PMC13079866; doi:10.1038/s41598-026-45654-3)
Supplement: Supplementary file 1 — Supplementary Material 1 [file 41598_2026_45654_MOESM1_ESM.docx]

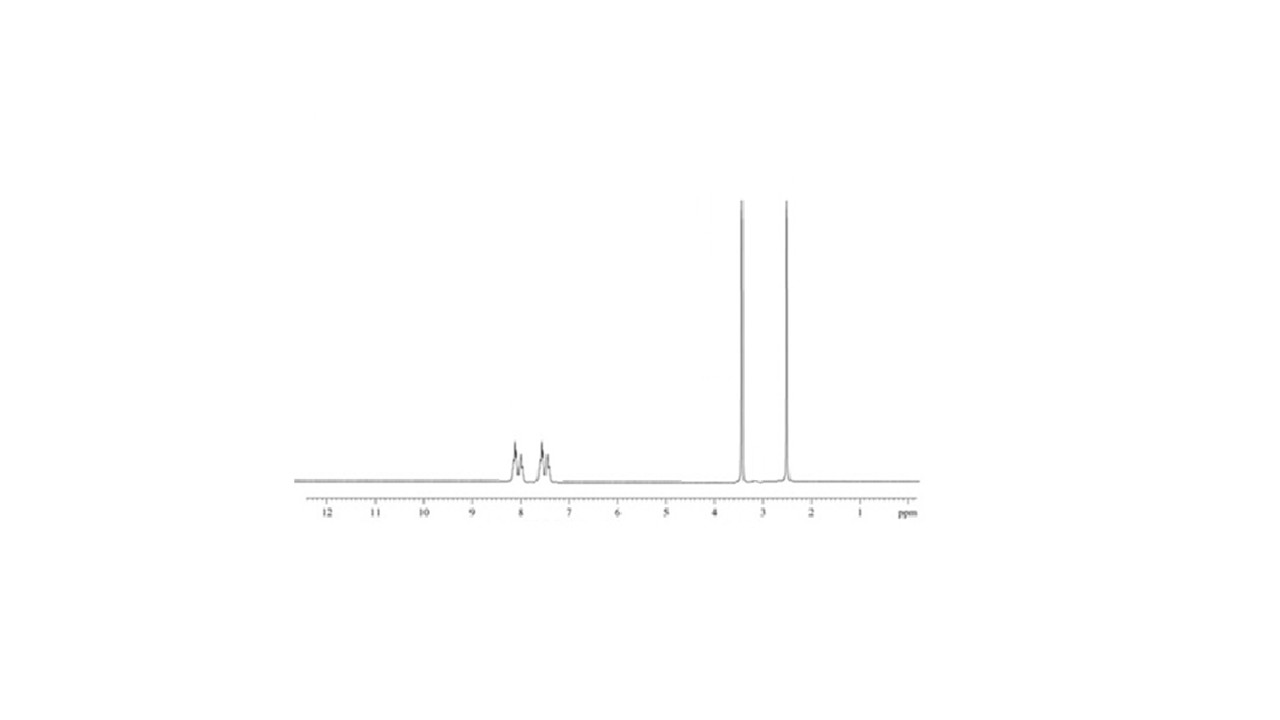


H-NMR of Compound 1


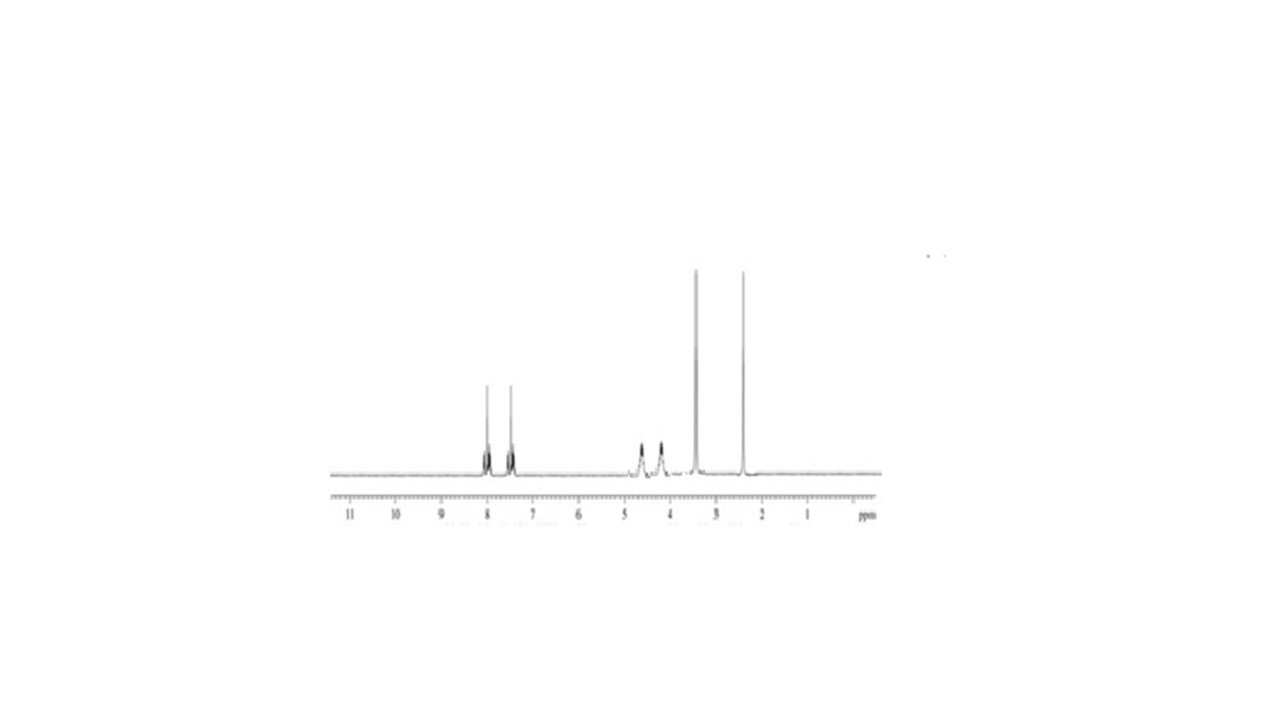


H-NMR of Compound 2


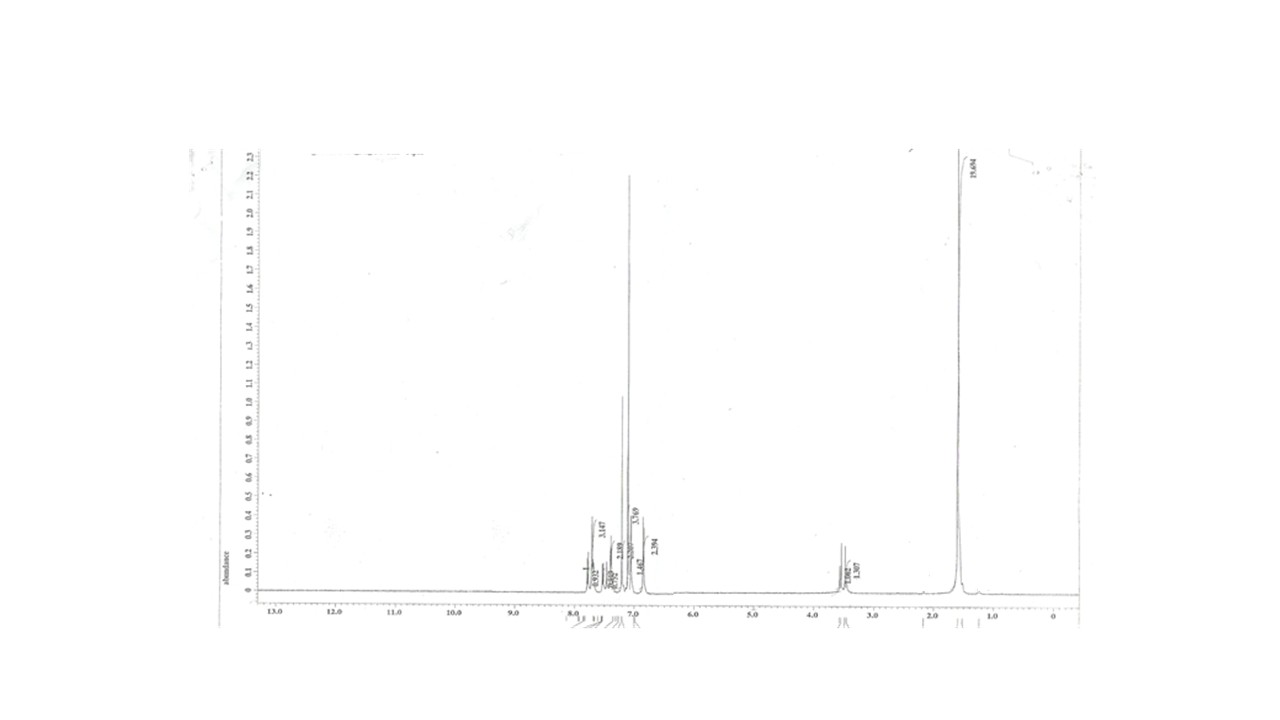


H-NMR of Compound 3


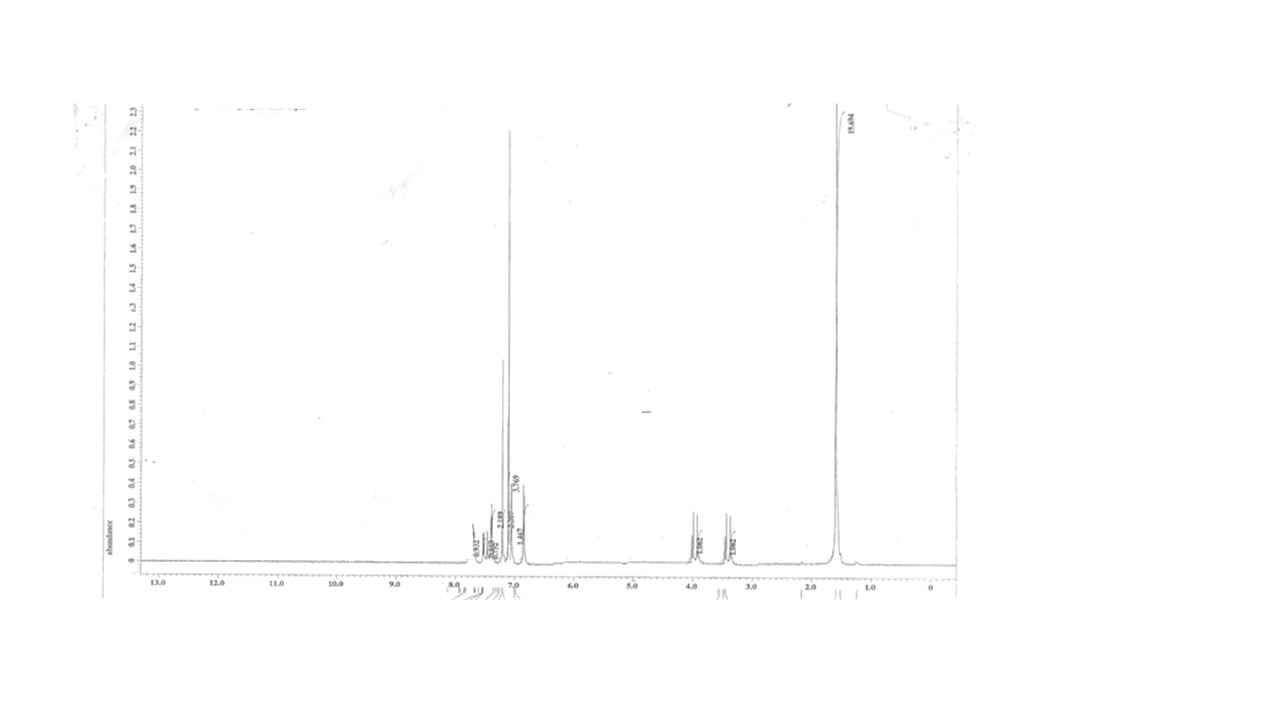


H-NMR of Compound 4


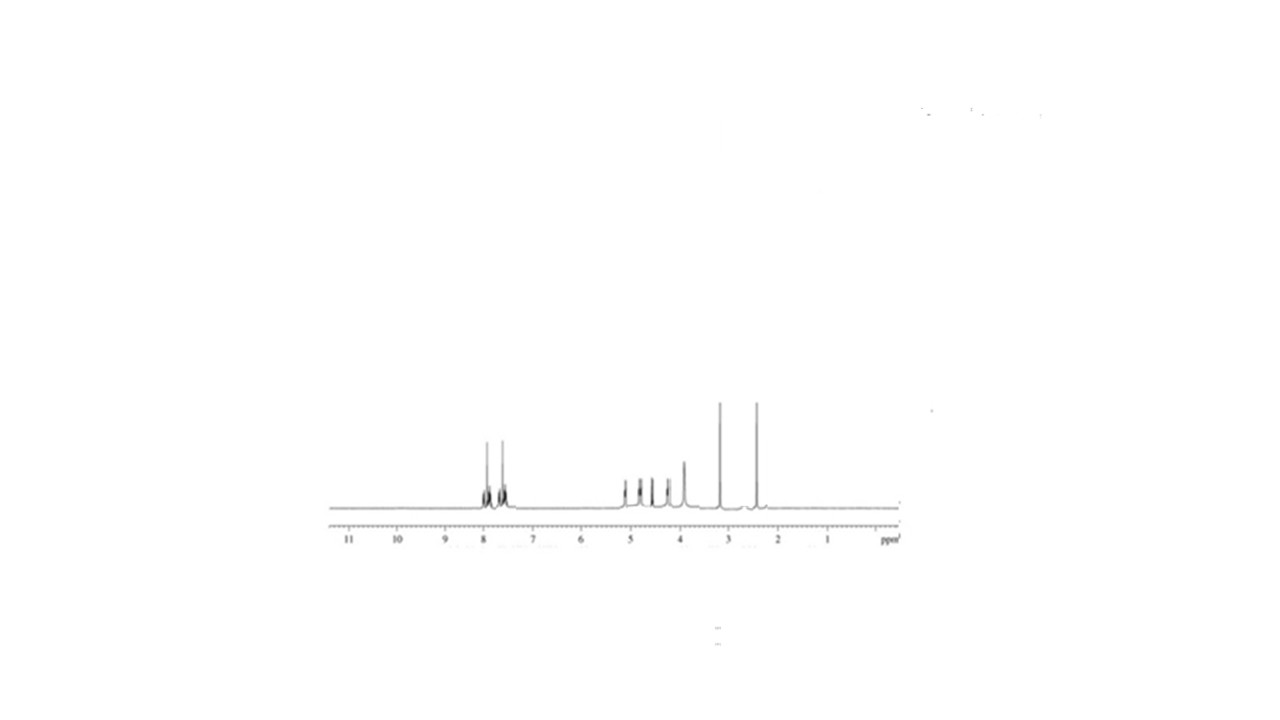


H-NMR of Compound 6a


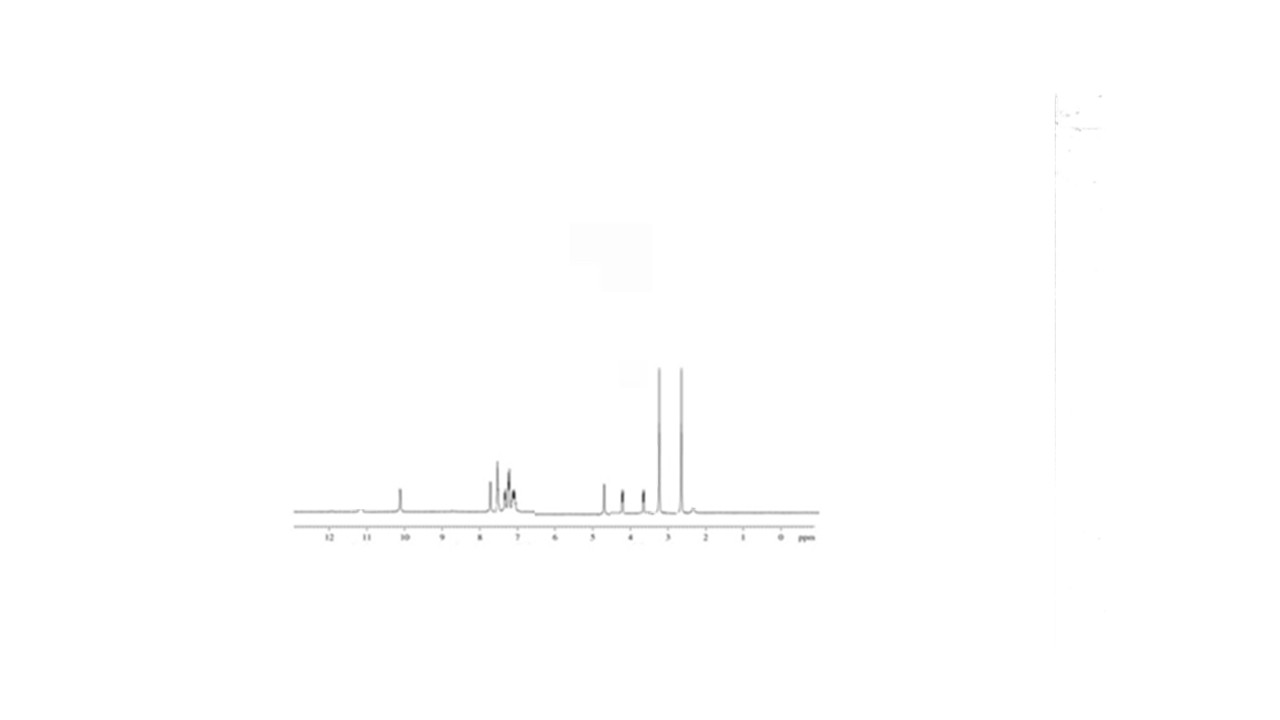


H-NMR of Compound 7


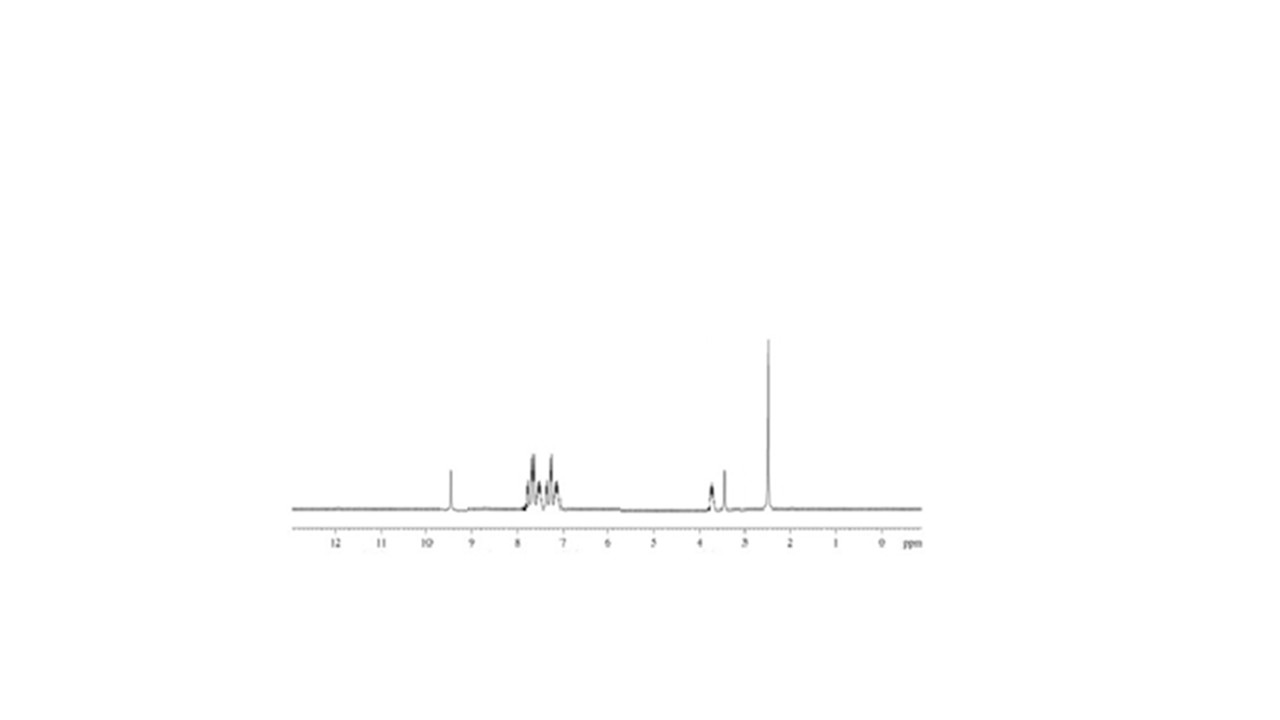


H-NMR of Compound 8


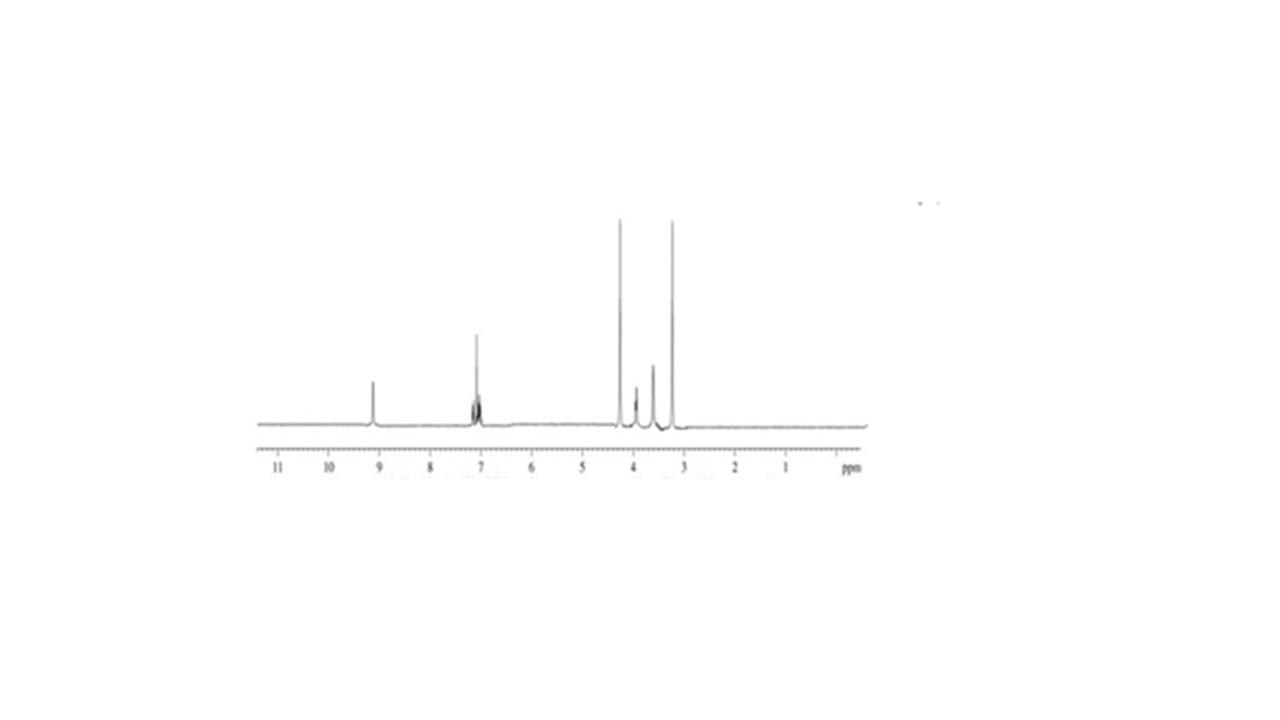


H-NMR of Compound9a


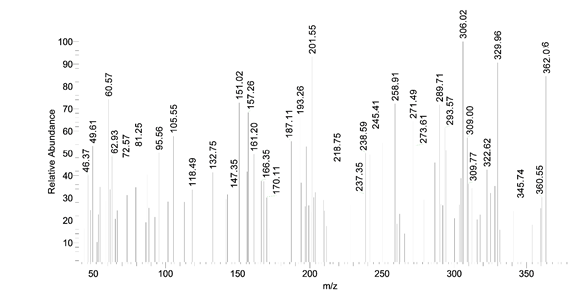

Fig. 1: Mass spectrum of compound 4


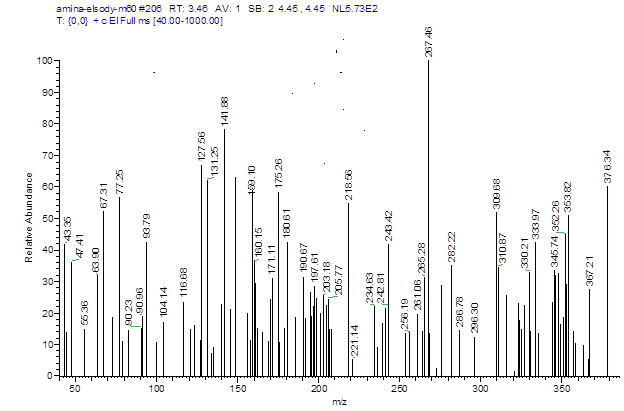

Fig. 2: Mass spectrum of compound 5


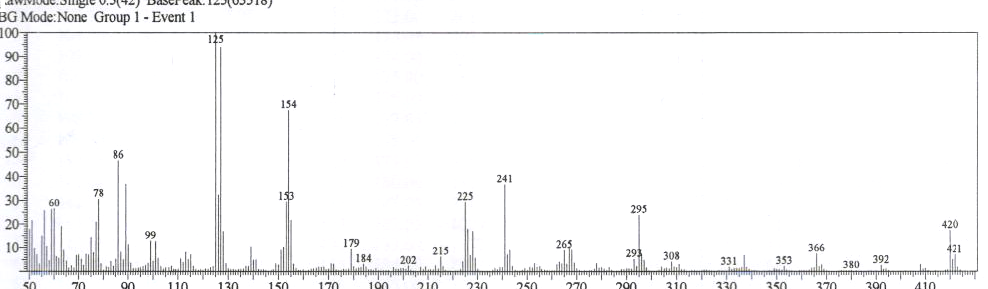

Fig. 3: Mass spectrum of compound 6b


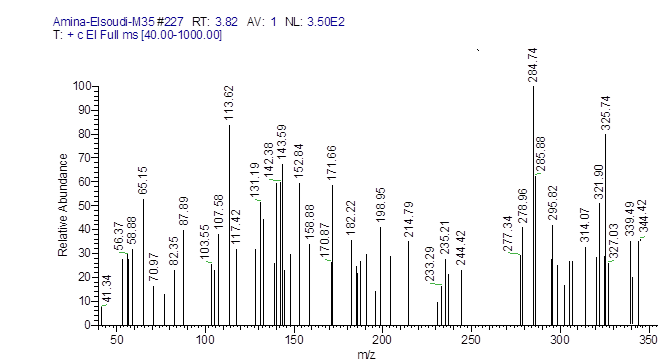

Fig. 4: Mass spectrum of compound 9a


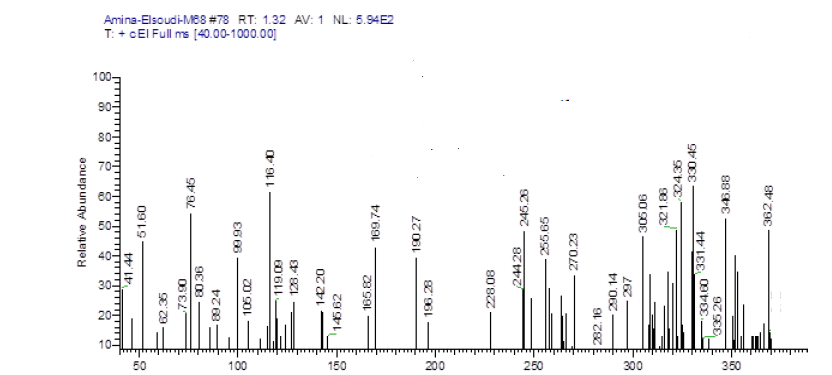

Fig.5: Mass spectrum of compound 11
